# Dexterous single sniffs for ethological active olfaction

**DOI:** 10.1126/sciadv.aed3610

**Published:** 2026-07-03

**Authors:** Mang Gao, John M. Barrett, Rita Fischer, Donald R. McCrimmon, Daniel W. Wesson, Minghong Ma, Gordon M. G. Shepherd

**Affiliations:** ^1^ Department of Neuroscience, Feinberg School of Medicine, Northwestern University, Chicago, IL, USA.; ^2^ Department of Pharmacology and Therapeutics, Florida Chemical Senses Institute, University of Florida College of Medicine, Gainesville, FL, USA.; ^3^ Department of Neuroscience, University of Pennsylvania Perelman School of Medicine, Philadelphia, PA, USA.

## Abstract

Rhythmic sniffing is considered intrinsic to active olfaction among terrestrial mammals. However, mice are known to briefly hold food under their nares while feeding, suggesting coordination of oromanual dexterity, breathing, and olfaction for nonrhythmic single sniffs. Here, we recorded kinematics and breathing as mice foraged and fed, finding that mice indeed, with clockwork-like dexterity and millisecond timing, synchronize a single inspiration with rapid head and hand movements. These solitary food sniffs are associated with abrupt resetting of the breathing rhythm, differ from stereotypical rhythmic sniffing, and exhibit behavioral modulations for different food properties. Olfactory and motor circuit manipulations demonstrate motor cortical dependence rather than reflexive neural control. Our study extends the concept of active olfaction to include this distinct form of complex motor-sensory coordination, features of which accord with the idea of discrete “snapshot” olfaction.

## Teaser

A discrete, motor cortex–dependent form of olfactory sampling uses millisecond coordination of hands, head, and breathing.

## INTRODUCTION

**Fig. 1.**
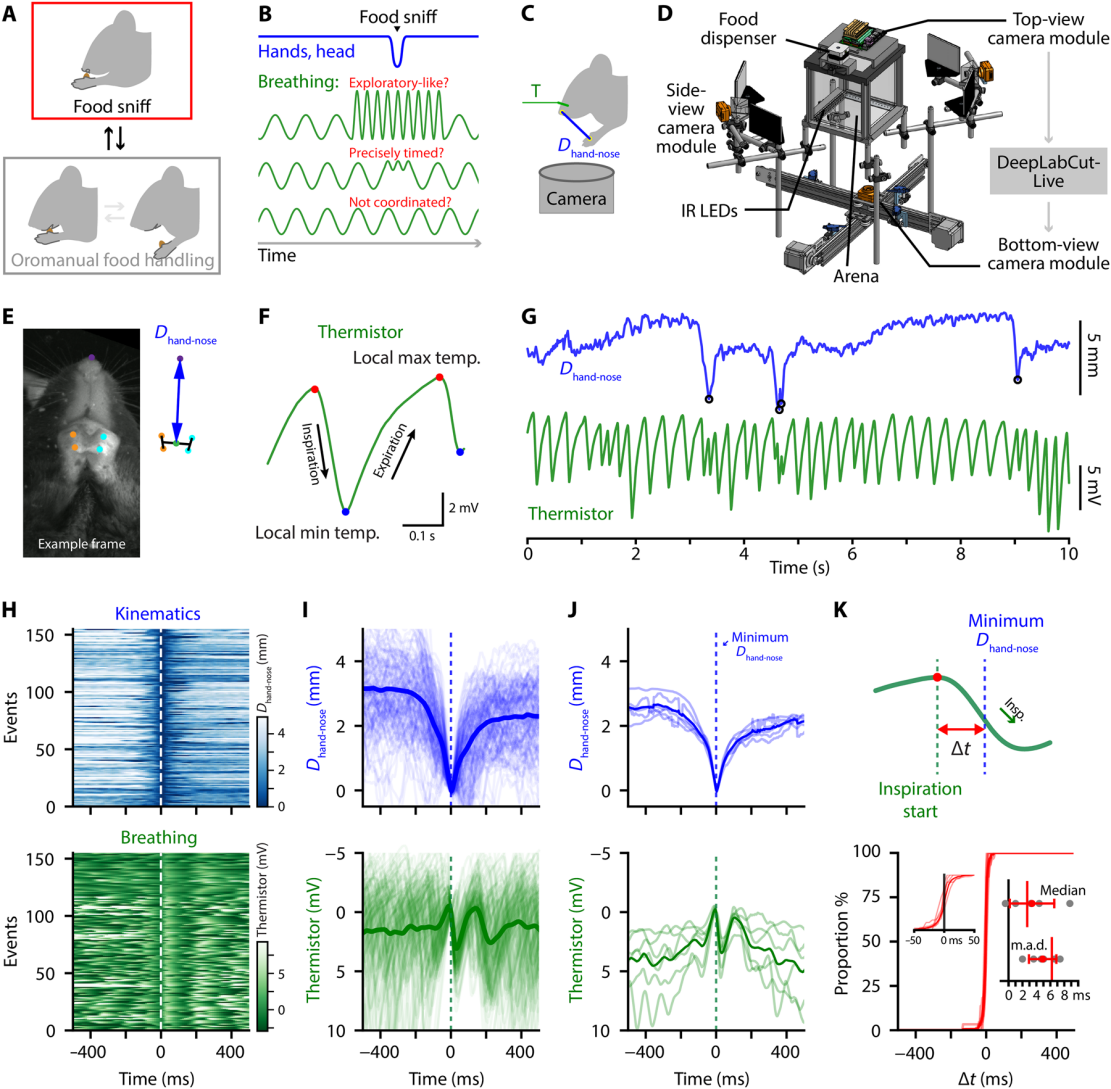
Food sniff kinematics synchronize with a single inspiration. (**A**) Food sniffs are distinct intermittent movements during oromanual food handling behavior. (**B**) Hypothetical models of breathing coordination with hand and head movements during food sniffs (see text). (**C**) Simultaneously recording kinematics (*D*_hand-nose_) with high-speed video and breathing signals by intranasal thermistor (T). (**D**) Behavioral recording system. The motorized zoomed-in bottom-view camera tracked the animal in real time, based on DeepLabCut-Live analysis of the top-view camera’s image, with additional cameras providing side views. IR, infrared. (**E**) Bottom-view video frame, marked with the keypoints used to calculate the hand-nose distance (*D*_hand-nose_). (**F**) Example voltage signal recording from the implanted thermistor. (**G**) Example recording, showing the *D*_hand-nose_ trace, with several detected food sniff events (circles), and the concurrent breathing signal. (**H**) Example recordings from one mouse of food sniff movements (top, blue traces) and corresponding breaths (bottom, green). Data were aligned by the time the *D*_hand-nose_ reached minimum and shown in order of acquisition. (**I**) Individual (thin lines) and average (thick) traces for the same data. Kinematic traces were normalized by subtracting the minimum-*D*_hand-nose_ value. (**J**) Average traces for six mice (thin lines), along with the grand average across mice (thick). (**K**) Top: Time difference (Δ*t*) between food sniff kinematics and breathing, calculated as the minimum-*D*_hand-nose_ time minus the start of the closest inspiration. Bottom: Cumulative distributions of Δ*t* for each mouse (thin lines) are plotted along with the overall average across mice (thick). Inset on left: Same data on a faster time base. Inset on right: The median (top) and m.a.d. (bottom) of Δ*t* for each mouse (gray circles) are plotted along with the overall averages across mice (red circles with error bars; mean ± SD) and the median of the average distribution (red vertical line).

“Dexterity” takes diverse forms in animal behaviors that rely on skilled movements of limbs and other body parts. Those involving hand-face interactions—oromanual dexterity—such as for feeding and grooming, are common in mammals and ethologically critical. In primates, hand-to-mouth and related handling movements are prominent and evolutionarily important (*1*, *2*). Among rodents, oromanual food handling is evolutionarily highly conserved (*3*). Mice manipulate and ingest food using a set of basic hand-jaw movements (*4*–*6*). They also intermittently bring the food to the nose, as if briefly wafting it by the nares (Fig. 1A) (*6*). These manually dexterous “food sniff” movements occurred frequently in freely moving but not head-fixed mice. Little else is known about these apparently common behaviors, including whether they even constitute bona fide sniffs; whether breathing is actually coordinated with hand movements; and, if so, exactly how.

Sniffing rhythms in mice are highly dynamic (*7*), and there are thus several hypothetical possibilities involving amplitude, rate, and/or timing modulations of the breathing rhythm around the time of the food sniff movements (Fig. 1B). Olfactory sniffing is considered inherently rhythmic—making it often difficult or impossible to distinguish from nonolfactory (e.g., mainly respiratory) breathing (*8*, *9*). Thus, mice might increase both the rate and amplitude of breathing over a prolonged span around the time of the sniff movements, similar to exploratory-type rhythmic sniffing commonly observed in rodents (*9*–*11*), or breaths might decrease in amplitude while increasing in rate, as observed in some odor discrimination tasks (*12*). Alternatively, given psychophysical evidence that single sniffs can suffice to discriminate odors (*13*–*16*), mice might time their hand movements, breathing cycle, or both, such that inspiration occurs when food is nearest to the nares. Such timing would need to be closely coordinated with the ongoing breathing rhythm (*17*). Such single sniffs, though not previously observed during ethological behavior, would accord with and bolster the hypothesis that olfactory sniffing is composed of discrete sensory “snapshots” (*8*, *13*). However, many other variations of these parameters and their combinations are possible, as is noncoordination (independence) of breathing and hand movements. To address these questions, we built a multimodal recording system combining robotically controlled high-speed videography of kinematics and simultaneous intranasal monitoring of breathing, and undertook in-depth quantitative investigation of food sniffing behavior in mice.

## RESULTS

### Recording food sniff kinematics and breathing in foraging mice

To record kinematics, we mounted a high-speed camera below a behavioral chamber on an *x*-*y* translation system, for a zoomed-in ventral view of the mouse through the chamber’s transparent floor (Materials and Methods; Fig. 1, C and D). The position of this bottom-mounted camera was controlled based on a top-mounted camera above the chamber, which provided a zoomed-out dorsal view of the mouse within the chamber. The video stream from this top camera was processed by DeepLabCut-Live (*18*) and a pretrained neural network to determine the *x*-*y* coordinates of the mouse’s head, which were used to dynamically position the bottom camera. The ventral-view videos were analyzed offline using DeepLabCut (*19*) to extract the coordinates of the nose tip and digits and calculate the hand-to-nose distance (*D*_hand-nose_) (Fig. 1E). Food sniff events were kinematically identified as rapid (~100 ms) dips in *D*_hand-nose_ (*6*). To record breathing, we implanted a thermistor in the nasal cavity (*20*), which transduced temperature changes into a voltage signal (Fig. 1F). This approach yielded simultaneous high-sensitivity recordings of breathing and behavior as freely moving mice sniffed the food in their hands during natural feeding (Fig. 1G and movie S1).

### Food sniff kinematics synchronize with a single inhalation

With this apparatus, we monitored hungry mice as they handled and consumed food items (*n* = 6 mice; range of 15 to 155 and mean of 57.3 food-handling events per mouse) and then used event alignment for visualization, averaging, and further analysis (Materials and Methods). Aligning the traces to the time when hands and food were closest to the nose (the minimum-*D*_hand-nose_) suggested near-perfect coincidence with the start of inspiration onset (Fig. 1, H to J, and movie S2). We calculated the time differences (Δ*t*) between the extrema of the kinematic and breathing data during the food sniffs (time of minimum-*D*_hand-nose_ minus the start time of the closest inspiration) (Fig. 1K). The median Δ*t* was extremely small, with inspiration onset leading the kinematic minimum by only ~3 ms on average (2.7 ms, calculated as the median of the overall average distribution; 3.4 ± 3.2 ms, calculated as the mean ± SD for *n* = 6 mice). Even more notably, Δ*t* values were extremely narrowly distributed, with a median absolute deviation (m.a.d.) of only ~6 ms (6.2 ms, calculated from the overall average distribution; 4.9 ± 2.0 ms calculated as the mean ± SD of individual mice) (Fig. 1K), a value far smaller than expected by chance (Materials and Methods; fig. S1). The estimate of the average Δ*t* (of ~3 ms), based on recordings made intranasally with a thermistor, is comparable to and therefore limited by the video rate (300 fps) and also does not take into account any latencies related to technical (e.g., frequency response properties of the detector), biomechanical (e.g., temporal dynamics of air flow through the nares and nasal passages), or neuromuscular (e.g., timing of respiratory control neurons and muscles) factors. However, the narrowness of the distribution of Δ*t* values should not be affected by such factors. These results show that kinematics and breathing are tightly coordinated during food sniffs, with millisecond-scale timing.

### The breathing rhythm adjusts abruptly around food sniffs

**Fig. 2.**
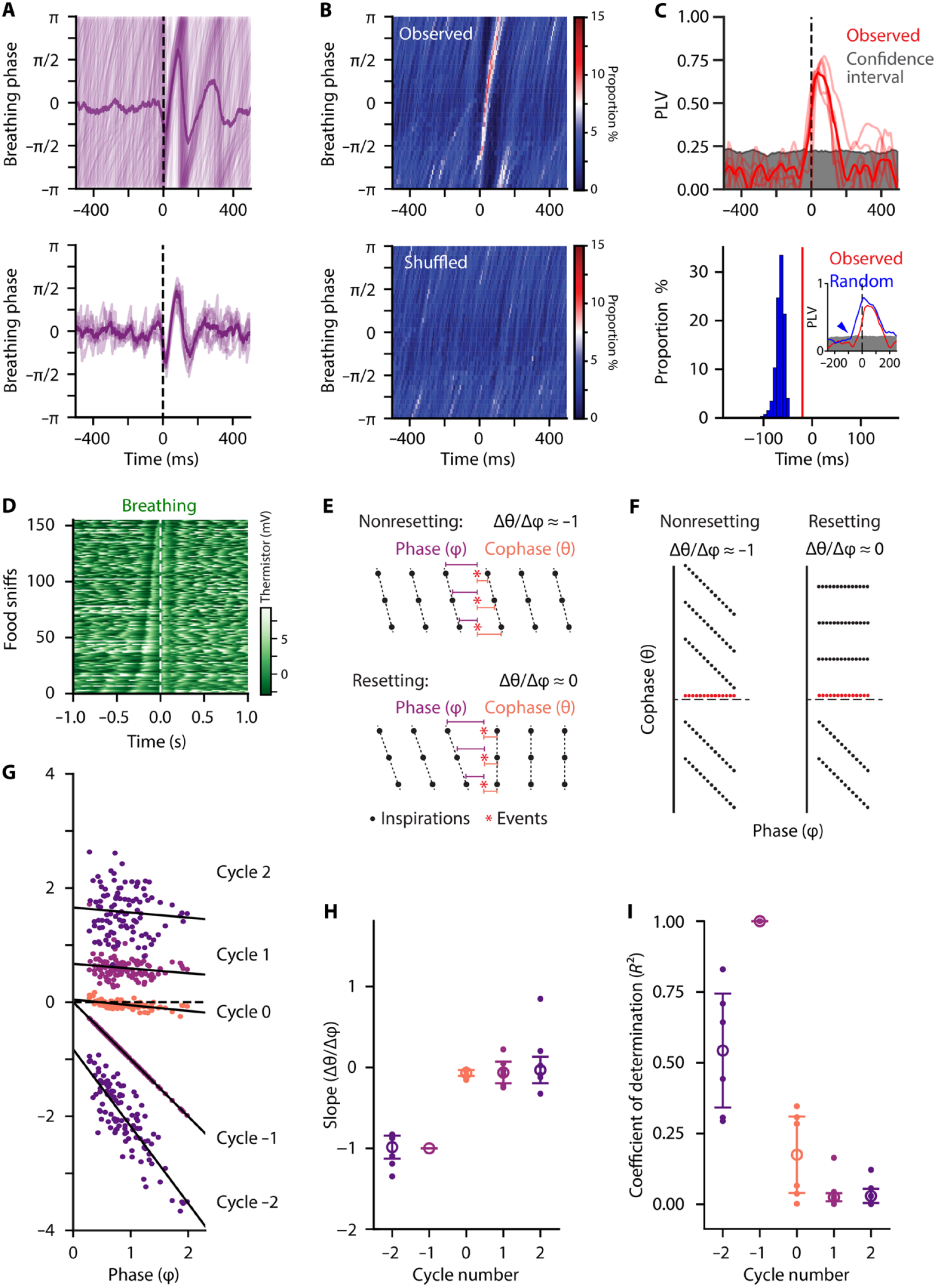
The breathing rhythm adjusts abruptly around food sniffs. (**A**) Top: Example (one mouse) of breathing phase during food sniff events (individual: thin traces; average: thick) were aligned to *D*_hand-nose_ minimum (0 ms). Inspiration start phase was set to −π. Bottom: Average breathing phase (thin lines, individual mice; thick lines, grand average, six mice). (**B**) Top: Average 2D distribution of breathing phase over time, normalized along phase (each time bin sums to 1). Bottom: Same, after shuffling. (**C**) Top: PLV (thin lines, individual mice; thick, group average). Gray, 99.8% confidence interval based on shuffling. Bottom: Onset times when the PLV of randomly chosen non–food-sniff breathing cycles (blue) and observed food-sniff breathing (red line) first exceeded shuffled data. Inset: Observed PLV and an example of the randomly chosen non–food-sniff inspiration (arrowhead, onset time). (**D**) Breathing recordings (from Fig. 1H) sorted by interval between food-sniff inspiration and preceding inspiration. (**E**) Nonresetting versus resetting breathing patterns (schematic). Dots, inspirations; asterisks, food sniffs. Phase (φ), time from food sniff to preceding inspiration; cophase (θ), time from food sniff to subsequent inspiration. For nonresetting (top), phase and cophase are inversely proportional. For resetting (bottom), cophase is constant, independent of phase. (**F**) Phase versus cophase for nonresetting and resetting (schematic). (**G**) Example phase (φ) versus cophase (θ) plot of food-sniff inspirations and preceding/subsequent inspirations (one mouse). Time zero was defined as the time of *D*_hand-nose_ minimum. Lines, linear regression of cophase over phase. (**H**) Regression slopes for each mouse, and grand medians (larger symbols ± m.a.d.), for each breathing cycle. Slope at cycle −1 is by definition −1. (**I**) Same, for the coefficients of determination (*R*^2^) of the regressions of phase-cophase data. The *R*^2^ of the regression for the data at cycle −1 is by definition 1.

How are food sniffs coordinated with the ongoing breathing rhythm? As an initial approach, we analyzed the extent of phase locking of breathing with the food sniff kinematics (same recordings as above). We obtained the instantaneous phase of each breath around the food sniff events (Fig. 2A) and computed the phase-time distribution (Materials and Methods; Fig. 2B). The breathing phase pattern was highly asymmetric around the event time, reaching high concentration from just before the *D*_hand-nose_ reached its minimum until well after. We quantified the average phase-locking value (PLV) of breathing during the food sniff events and compared these data to shuffled data. We computed a 99.8% confidence interval of PLV along time with the shuffles. The PLV was significantly higher than the shuffled data starting at 26 ± 7 ms before the event time and lasting until 160 ± 7 ms after (Fig. 2, B and C). For comparison, we performed the same analysis using randomly chosen non–food-sniff inspirations that occurred close in time (±0.5 s) to food-sniff inspirations (Materials and Methods; fig. S2). This showed that the PLV onset time of the observed data (26 ms) was clearly later than expected if the food sniff was timed to the ongoing breathing rhythm (67 ± 7 ms, *P* = 10^−4^, based on shuffling) (Fig. 2C). These results show that phase locking of the breathing rhythm starts immediately before the food is closest to the nares and then continues for slightly more than one breath cycle.

This phase locking, together with the synchronization of inspiration and kinematics, raises the question of whether the breathing rhythm also resets around food sniffs and, if so, over what timescale. We adapted the concept and quantification method of breathing rhythm resetting from previous literature (*21*, *22*). If resetting happens at various phases of the ongoing breathing cycle, the subsequent inspiration timing should stay constant. Sorting the *D*_hand-nose_ minimum-aligned breathing signals by the interval from the preceding inspiration to the food-sniff inspiration showed that the food sniff breathing cycle durations appeared similar despite different durations of the preceding cycle (Fig. 2D). To evaluate this, we quantified the phase (φ) as the time interval from the inspiration preceding the peri–food-sniff inspiration to the *D*_hand-nose_ minimum, and the cophase (θ) as the time from the *D*_hand-nose_ minimum to the surrounding inspirations (preceding and following). We divided these by the median breathing cycle duration for each video to normalize the phase and cophase (Materials and Methods). If food sniffs are causally independent of the breathing rhythm, then this approach would allow us to distinguish between two hypothetical models, in which events either do or do not reset the breathing rhythm (Fig. 2, E and F). These two models predict distinct plots of phase versus cophase. In the resetting model, the cophase stays constant as the phase varies, resulting in a Δθ/Δφ ≈ 0. A third possibility (although suggested against by the PLV analysis; Fig. 2C) is that food sniffs are simply timed to the existing rhythm without adjustment (i.e., nonindependent), which theoretically predicts a range of possible slopes, including 0 (Materials and Methods).

Plotting the experimentally observed phase and cophase showed close agreement with the resetting model (Fig. 2G). We analyzed these data using linear regression and calculated the Δθ/Δφ of each breathing cycle in the peri–food-sniff interval (same recordings as above). The Δθ/Δφ changed from an initial value close to −1 (−0.98 ± 0.14, median ± m.a.d., at the inspiration before the preceding inspiration) to a value of ~0 when the food sniff occurred (−0.07 ± 0.04, Wilcoxon signed-rank test, *W* = 0, *P* = 0.031) and remained low thereafter (−0.06 ± 0.13, first inspiration after the food sniff; −0.03 ± 0.16, second inspiration after the food sniff) (Fig. 2H). The coefficient of determination (*R*^2^; gauging correlation strength) changed from initially high values (0.54 ± 0.20, median ± m.a.d., at the inspiration before the preceding inspiration) to low values when the food sniff occurred (0.17 ± 0.13, *W* = 0, *P* = 0.031) and remained low thereafter (0.02 ± 0.01, *W* = 0, *P* = 0.031, first inspiration after the food sniff; 0.03 ± 0.02, *W* = 0, *P* = 0.031, second inspiration after the food sniff) (Fig. 2I). To assess how well these data fit each model (nonindependent, nonresetting, and resetting), we performed shuffled controls to calculate the expected distributions of Δθ/Δφ and *R*^2^ under each (Materials and Methods; fig. S3). As expected, the nonresetting model was a poor fit to the data (Δθ/Δφ: *P* < 0.001, *R*^2^: *P* < 0.001). The nonindependent model predicted a similar *R*^2^ (*P* = 0.31) but a more positive Δθ/Δφ slope than shown in the real data (*P* = 0.04). Last, the observed slope (*P* = 0.84) and *R*^2^ (*P* = 0.99) were both plausible under the resetting model. Thus, the nonresetting and nonindependent models do not adequately explain the observed data, whereas the resetting model cannot be ruled out on the basis of the available data.

Relevant to these phase-related measurements, prior work (*23*) has shown that the expiratory phase of air flow when recorded by thermocouple (in contrast to pressure transducer) can reflect a passive component (rather than forced exhalation, as with a sneeze), a consideration likely to apply to the thermistor-based air flow measurement used here. However, although our measurements do not distinguish between active exhalation and passive temperature changes (such as during pauses), this would not affect estimates of inspiration onset. Collectively, these results show that the breathing rhythm adjusts abruptly around food sniffs, providing further evidence of the precise coordination between forelimb kinematics and breathing.

### Hand and head movements are also tightly synchronized during food sniffs

**Fig. 3.**
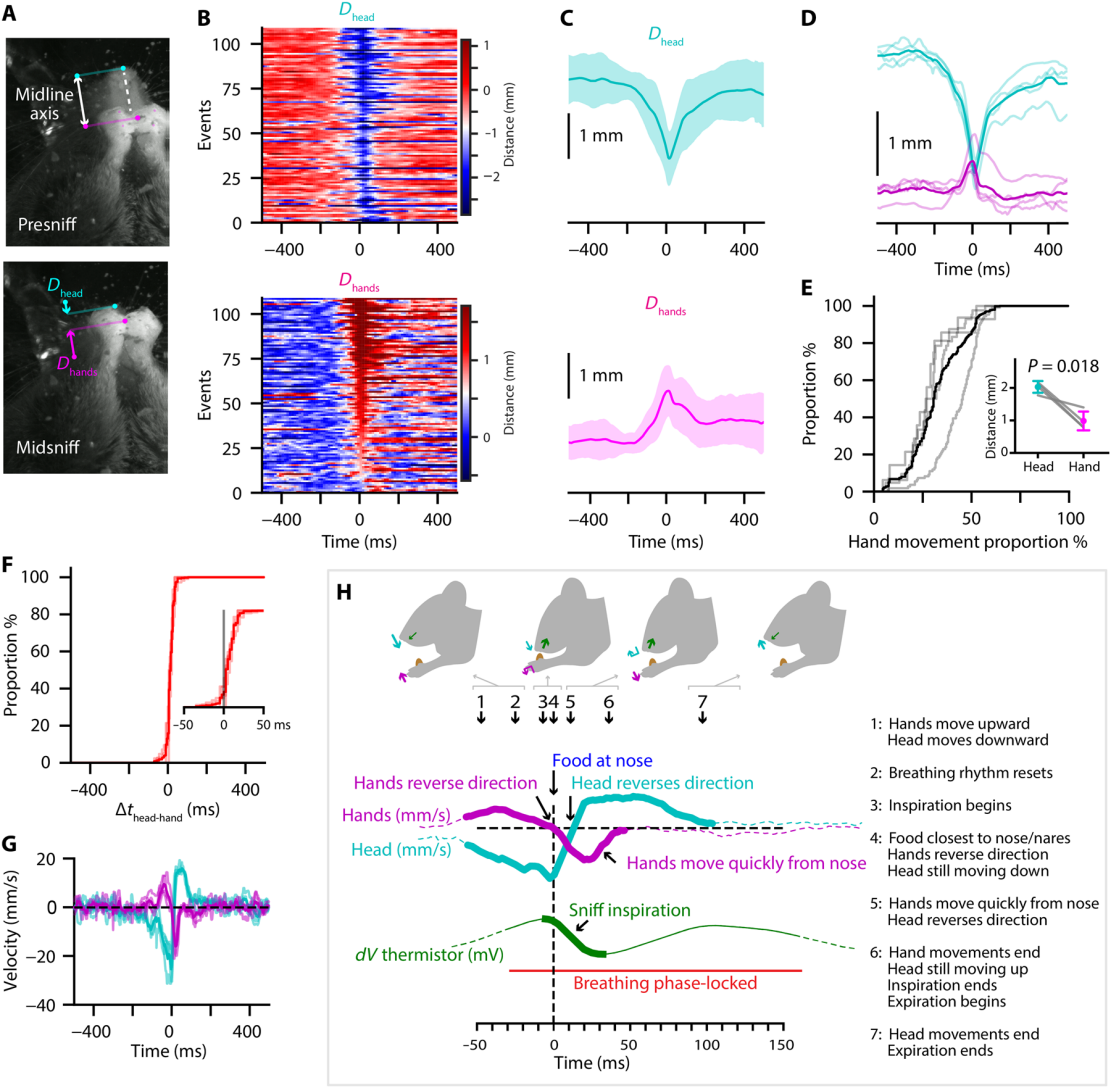
Hand and head movements are also tightly synchronized during food sniffs. (**A**) Approach for measuring the hand and head components of *D*_hand-nose_, along the midline (sagittal) axis (top). Movements of the head (based on the nose tip keypoint) and the hands (average of right and left hands) along this axis were tracked, relative to their baseline positions before and after the sniff events, as shown in these frames from just before (top) and during (bottom) a kinematic food sniff event. (**B**) Example recording from one mouse of the head (*D*_head_, top) and hand (*D*_hands_, bottom) components of longitudinal movements of food sniff movements, sorted by the hand/(hand+head) ratio. Traces were aligned by the time the *D*_hand-nose_ reached minimum. (**C**) Average trajectories for the head (top) and hand (bottom) components of *D*_hand-nose_ (with SD) for the same example data. (**D**) Average trajectories of the head and hand components of *D*_hand-nose_, for individual mice (*n* = 4; thin lines), along with the grand average (thick lines). (**E**) Cumulative distributions of the hand movement proportion [hand/(hand+head)] of food sniff movements for individual mice (gray) and averaged across mice (black). Inset: Average maximum distance the head (cyan) and hand (magenta) traveled during food sniffs (with SD). Gray lines, medians of individual mice. (**F**) The cumulative distributions of Δ*t*_head-hand_, calculated as the time between the extrema of the head and hand movements (*t*_head_−*t*_hand_), plotted for each mouse (thin lines) along with the overall average across mice (thick line). Inset: Same data on a faster time base. (**G**) Average velocities [first derivatives of the distance trajectories in (D)] of the head (cyan) and hand (magenta) components of *D*_hand-nose_. (**H**) Summary of key features of hand-head-breath coordination during food sniffs. The traces shown are the overall average traces shown in previous plots and figures.

The foregoing analysis considered the kinematic component based on the hand-nose distance, but a closer look at food sniff events in the videos suggested that the “manual” food sniffs also involve coordinated movements of the head, which dipped downward as the hands moved upward and forward to transport the food toward the nose (movie S3). We therefore analyzed the kinematics to determine the separate contributions of head and hand movements (Materials and Methods; Fig. 3A). As shown in the example plots (Fig. 3, B and C) and as borne out in the average recordings across mice (Fig. 3D), the head and hand movements appeared to be tightly synchronized, with broadly similar trajectories of opposite sign.

One obvious asymmetry, however, was a difference in movement amplitudes, which were overall twice as large for head than for hand trajectories (head: 2.0 ± 0.3 mm, hands: 1.0 ± 0.2 mm, mean ± SD of medians, *n* = 4 mice, *P* = 0.018, *t* test) (Fig. 3E, inset). Similarly, head movements also contributed more to the overall *D*_hand-nose_ trajectory during sniff events (71 ± 1% head versus 29 ± 1% hands) (Fig. 3E). This may be why food sniffs are infrequent in head-fixed mice (*6*).

Focusing on the temporal properties, we compared the timing of the extrema of the head and hand trajectories, which confirmed tight synchronization; the inflection of the head trajectory (*t*_head_) slightly trailed that of the hands (*t*_hand_), with Δ*t*_head-hand_ of only 15.2 ± 9.4 ms (median ± m.a.d., *t*_head_−*t*_hand_) (Fig. 3F). In addition, the velocity profiles of the trajectories, although overall broadly similar and mirrored (as expected), were generally larger and longer for the head than for the hands (Fig. 3G). Hand movements immediately after the peak of the sniff movement were rapid and brief, both in comparison to hand movements before the peak and to head movements before and after the peak.

Combined plotting, on a faster time base, of the same overall average velocity profiles of the head and hand trajectories (from Fig. 3G) together with the overall average breath activity (from Fig. 1J) clarifies the intricate and rigidly orchestrated high-speed sequence of events involved in food sniffs (Fig. 3H). In the initial phase, movements of the head and hands toward each other begin to bring the food toward the nose. Relative to “peak sniff” at time 0 ms (minimum-*D*_hand-nose_), at about −25 ms, the breathing phase-locks to the kinematics, during end-expiration, and the sniff inspiration starts at about −3 ms. During this immediate presniff period, the hand movements are slowing but the head movements remain fast. At 0 ms, with the sniff just initiated and with food closest to the nares, the hands reverse direction. The head abruptly starts to slow down, but continues moving downward for another ~10 ms before reversing direction. Meanwhile, by ~40 ms postpeak, the hands have briskly retracted the food away from the nares, and the sniff inspiration has ended as the expiration begins. The head returns to its presniff posture by ~100 ms, and breathing returns to its baseline rhythm shortly after. Thus, although the overall trajectory of *D*_hand-nose_ appears symmetric in time around the peak of the sniff event, the kinematics of the hand and head components and the sniff-related breathing components are sharply asymmetric in time around the peak of the sniff event and instead tightly coordinated in a rapid asynchronous sequence.

### Food sniffs differ from other modes of sniffing

**Fig. 4.**
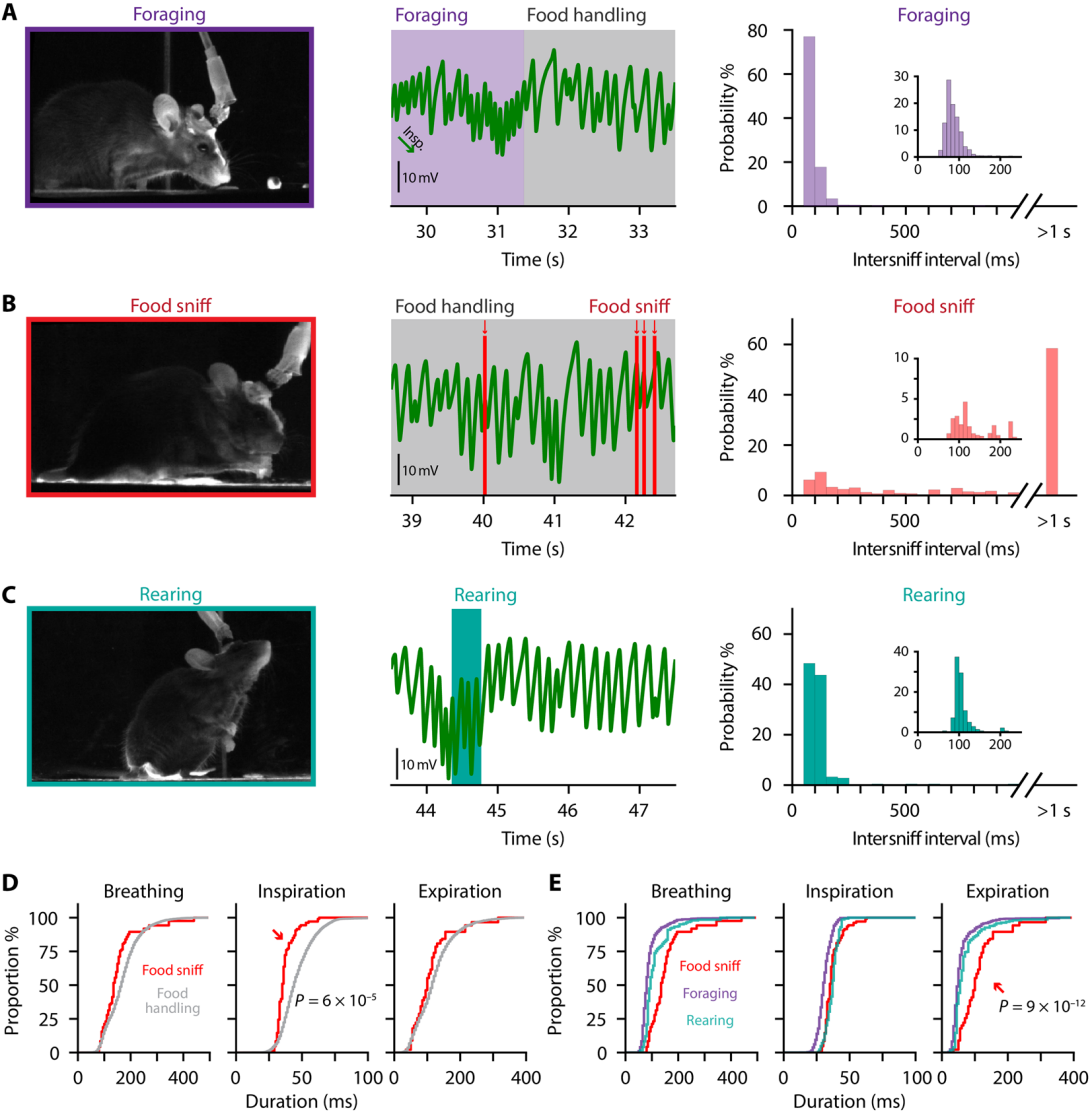
Food sniffs differ from other modes of sniffing. (**A**) Foraging-related sniffs. Left: Example movie frame showing typical posture during foraging-related behavior between when food was presented and found. Center: Breathing signal (green trace) during an epoch of foraging behavior (purple shading) followed by an epoch of food handling (gray shading). Right: Histogram of intersniff intervals [overall average histogram across mice (*n* = 6 mice)]. Inset: Histogram of the same data, on a faster time base with smaller time bins. (**B**) Same, for food sniffs (*n* = 9 mice). Example trace from later in the same recording as in (A). Vertical red lines and arrows indicate food sniff events. (**C**) Same, for rearing-related sniffs (*n* = 6 mice). Example trace from later in the same recording as in (B). An epoch of rearing is indicated by aquamarine shading. (**D**) Mean cumulative distributions (average of *n* = 6 mice) of the durations of breathing (left), inspiration (middle), and expiration (right), during food sniffing (red) and general food handling (gray). Note the relatively short durations of food-sniff inspirations (arrow). (**E**) Similar to (D), but for food sniffing (red), foraging (aquamarine), and rearing (purple). The relatively long durations of food sniff expirations (arrow).

Our recordings of freely moving mice also captured bouts of rhythmic sniffing associated with rearing and foraging behavior as mice moved about within the chamber before and after handling food (Materials and Methods; Fig. 4, A to C, and movie S4). Foraging epochs were identified as events before or after food handling when the head was angled downward, close to the chamber floor, searching for food. Foraging-related sniffing was characterized by sustained ~12-Hz breathing (11.6 ± 1.7 Hz, 86 ± 16 ms, median ± m.a.d., *n* = 6 mice) (Fig. 4A). Rearing epochs were identified as events when the mouse vertically extended the body, with bipedal stance and head angled up, appearing to sniff the air. Rearing-related sniffing was characterized by ~10-Hz breathing (9.7 ± 1.1 Hz, 103 ± 19 ms) (Fig. 4C). In contrast, during food handling epochs, the overall breathing rate (excluding food sniffs) was lower, at ~6 Hz (5.9 ± 1.8 Hz, 170 ± 44 ms), with food sniffs occurring mainly as discrete events with mostly irregular timing (Fig. 4B). “Breathing rate” refers to the number of respiratory cycles per unit of time, whereas “food sniff rate” refers to the number of occurrences of food sniff events per unit of time. Intersniff intervals (histograms in Fig. 4, A to C) were generally very short for both foraging- and rearing-related sniffing, consistent with the runs of rhythmic high-frequency breathing during these behaviors, but were generally very long for food sniffs, consistent with their mostly intermittent and aperiodic occurrence during food handling. However, food sniffs did occasionally occur as short runs of doublets or triplets, with intersniff intervals similar to those observed during foraging and rearing. In contrast to these occasional brief bursts of rhythmic food sniffs, we did not observe cases where food was held statically at the nose over multiple breathing cycles.

We further analyzed the inspiratory and expiratory phases of the different sniff types. First, focusing on the breathing pattern during food handling, we found that the duration of the inspiratory phase was shorter for food sniffs [37 ± 6 ms, median ± m.a.d., *D* = 0.46, *P* = 6 × 10^−12^, Kolmogorov-Smirnov (KS) test] compared to other breaths during general food handling (45 ± 8 ms) (Fig. 4D). In contrast, expiratory durations were essentially identical. Second, focusing on the other forms of sniffing, we found that expiration durations for food sniffs (105 ± 23 ms) were notably longer in duration compared to both rearing- (54 ± 14 ms, *D* = 0.49, *P* = 3.4 × 10^−10^, KS test) and foraging- (63 ± 23 ms, *D* = 0.60, *P* = 1.2 × 10^−17^, KS test) type sniffs (Fig. 4E). In contrast, inspiration durations were similarly fast for food sniffs and rearing sniffs (39 ± 3 ms, *D* = 0.27, *P* = 0.192, KS test) and only slightly shorter for foraging sniffs (32 ± 3 ms, *D* = 0.66, *P* = 1 × 10^−11^, KS test). Although, as noted above, our thermistor-based measurements do not distinguish between active exhalation and passive temperature changes, this should not affect the estimation of the total duration of the expiration phase (defined as the phase between two inspirations).

Food sniffs thus differ from these other forms of sniffing, not only in their discreteness and overall rate but in the durations of the inspiratory and expiratory phases. A shortened inspiratory phase is common to all three forms of olfactory sniffing, but, whereas the food-sniff inspiration is followed by a longer expiratory duration, the high breathing frequencies during rearing and foraging sniffing reflect shorter expiration as well as inspiration.

### Food sniffs are behaviorally modulated

**Fig. 5.**
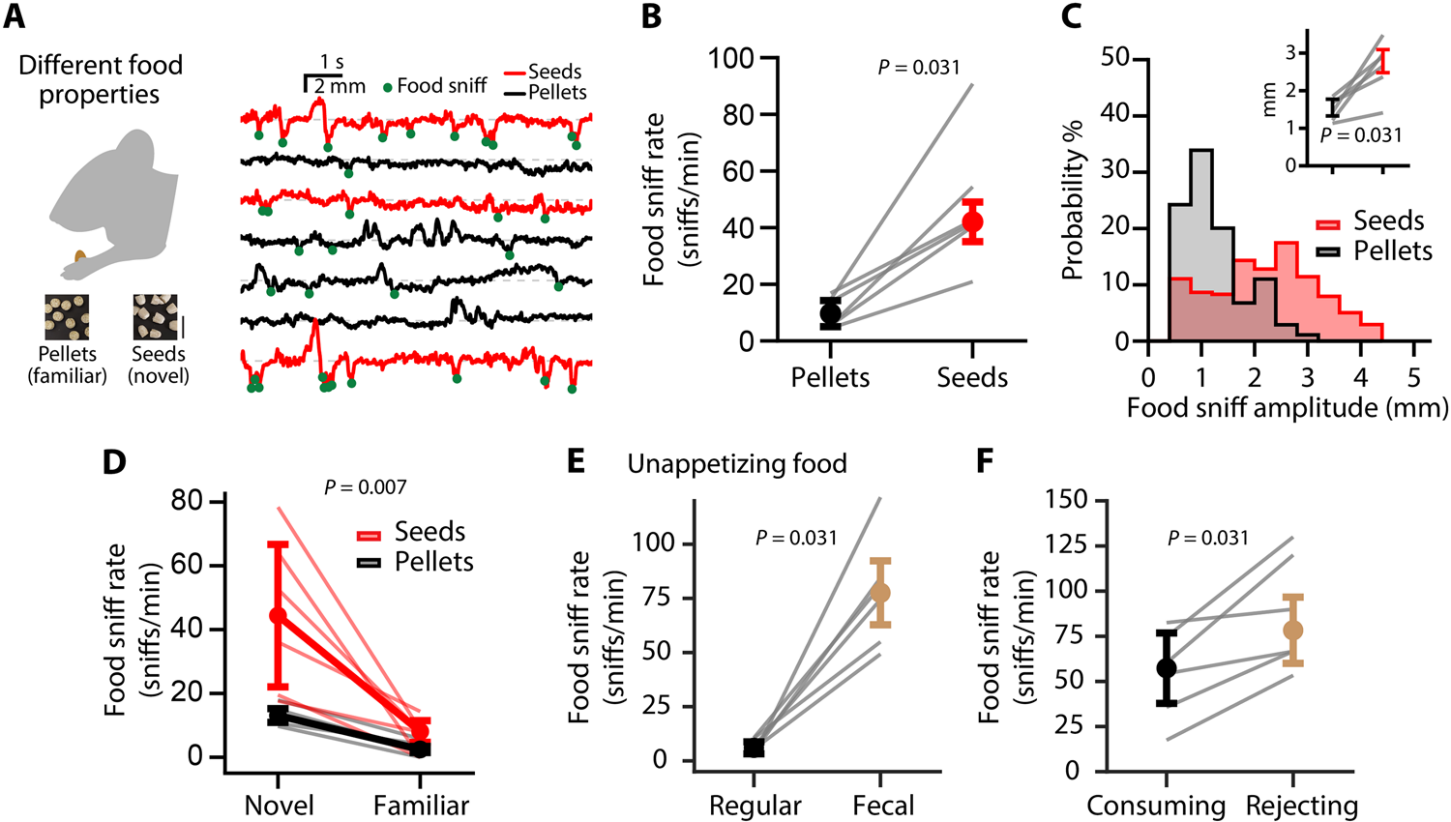
Effects of food properties on food sniffs. (**A**) Left: Mice received familiar pellets or unfamiliar sunflower seeds. Scale bar, 10 mm. Right: Example *D*_hand-nose_ traces handling pellets or seeds. Dots mark food sniff events. (**B**) Food sniff rate when mice were handling pellets or seeds (lines, individual mice; symbols and error bars, median ± m.a.d. across mice, *n* = 6 mice, Wilcoxon signed-rank test). (**C**) Food sniff amplitudes for familiar pellets and unfamiliar seeds (overall averages across mice). Inset: Food sniff amplitudes for pellets or seeds, for individual mice and group averages. (**D**) In an additional experiment, mice that were naive to both seeds and pellets were given either of these and then retested after familiarization. Plot shows the food sniff rate at first exposure to the new food (novel) and on the third day of exposure (familiar), for pellets (gray lines) and seeds (red lines). Lines, individual mice; symbols, overall median ± m.a.d.; data are from six mice that were first given pellets for 3 days and then seeds for 3 more days (two-way repeated measures ANOVA, both main effects and interaction were significant; see tables S1 and S2). (**E**) Food sniff rates of mice while handling regular or unappetizing food items. “Regular” food consisted of food balls crafted from ground-up standard food pellets. “Fecal” food was the same, but with a core of the mouse’s own fecal material. Lines, individual mice; symbols, overall median ± m.a.d.; *n* = 6 mice. (**F**) For the same experiment, food sniff rates while handling the food-coated fecal balls were calculated during the time when mice were initially consuming the item, compared to the time interval just before rejecting the item (starting 2 s prior to the rejection).

Prior studies show modulation of sniff vigor for different odorant types and properties (*24*), and breathing is also influenced by motivational state and experience. We therefore explored whether food sniffs exhibit behavioral modulation. If mice use food sniffs to actively sense the food while feeding, such behavior could be affected by properties of the food items such as their novelty versus familiarity. To test this possibility, mice were first familiarized to one food type by giving them only small food pellets (different in size but similar in composition to the standard laboratory diet used in the vivarium) during the two initial behavioral habituation sessions, as in the previous experiments. Then, sunflower seeds, a novel food type, were given in subsequent behavioral sessions, alternating with the familiar pellets (movie S5). As shown in the example traces of *D*_hand-nose_ recorded on the first day of exposure to the new food item (Fig. 5A), food sniffs were more frequent (42.0 ± 4.6 events/min versus 9.6 ± 7.0 events/min, median ± m.a.d.) (Fig. 5B). Food sniffs were also larger in amplitude when mice handled the newly introduced seeds compared to the familiar pellets (2.8 ± 0.3 mm versus 1.5 ± 0.2 mm) (Fig. 5C).

To further assess the extent to which these differences in food sniff behavior reflect food novelty/familiarity, type, or both, we repeated the experiment but, for both food types, compared the food sniff rates on the first days (when food items were novel) versus 2 days later (when they were familiar); we also reversed the order, presenting the seeds first and the pellets second (Fig. 5D). Both food types showed similar patterns of initially higher rate and amplitude of food sniffs, which fell over subsequent days to lower levels [two-way repeated measures analysis of variance (ANOVA), *P* = 0.007; tables S1 and S2]. In addition, food sniff rates were generally higher for seeds than pellets (*P* = 0.017), possibly reflecting the similar composition of the pellets and regular mouse food. These results thus demonstrate that food sniffing behavior is not rigidly stereotyped but actively adjusted for different food properties, including not only the type of food but also its novelty.

These results do not, however, address whether food sniffs are also associated with subsequent changes in food consumption, since mice went on to consume the food in all cases. To explore this, we considered that before handling the food, mice must first localize it within the chamber (in the low-light environment) using sustained rhythmic olfaction, during which they make the initial decision whether or not to pick it up and start feeding on it. Thus, the subsequent single food sniffs that occur during food handling may serve as a quick “smell check” to assess whether the food is still palatable. To test this, we presented mice with custom-made food balls consisting either of regular food pellet material only (control food balls) or containing a core composed of the mouse’s own fecal droppings (i.e., food-coated fecal balls). Mice sniffed at higher overall rates when handling the food-coated fecal food balls compared to the control food balls (6.0 ± 2.7 events/min versus 77.6 ± 14.8 events/min, median ± m.a.d., *n* = 6, Wilcoxon signed-rank test, *W* = 0, *P* = 0.031) (Fig. 5E). Furthermore, they discarded them prior to finishing them (movie S6), and their sniff rates increased shortly before doing so (78.3 ± 19.5 events/min versus 57.3 ± 18.3 events/min, median ± m.a.d., *n* = 6, Wilcoxon signed-rank test, *W* = 0, *P* = 0.031) (Fig. 5F).

### Food sniffs are largely unaffected by olfactory manipulations

**Fig. 6.**
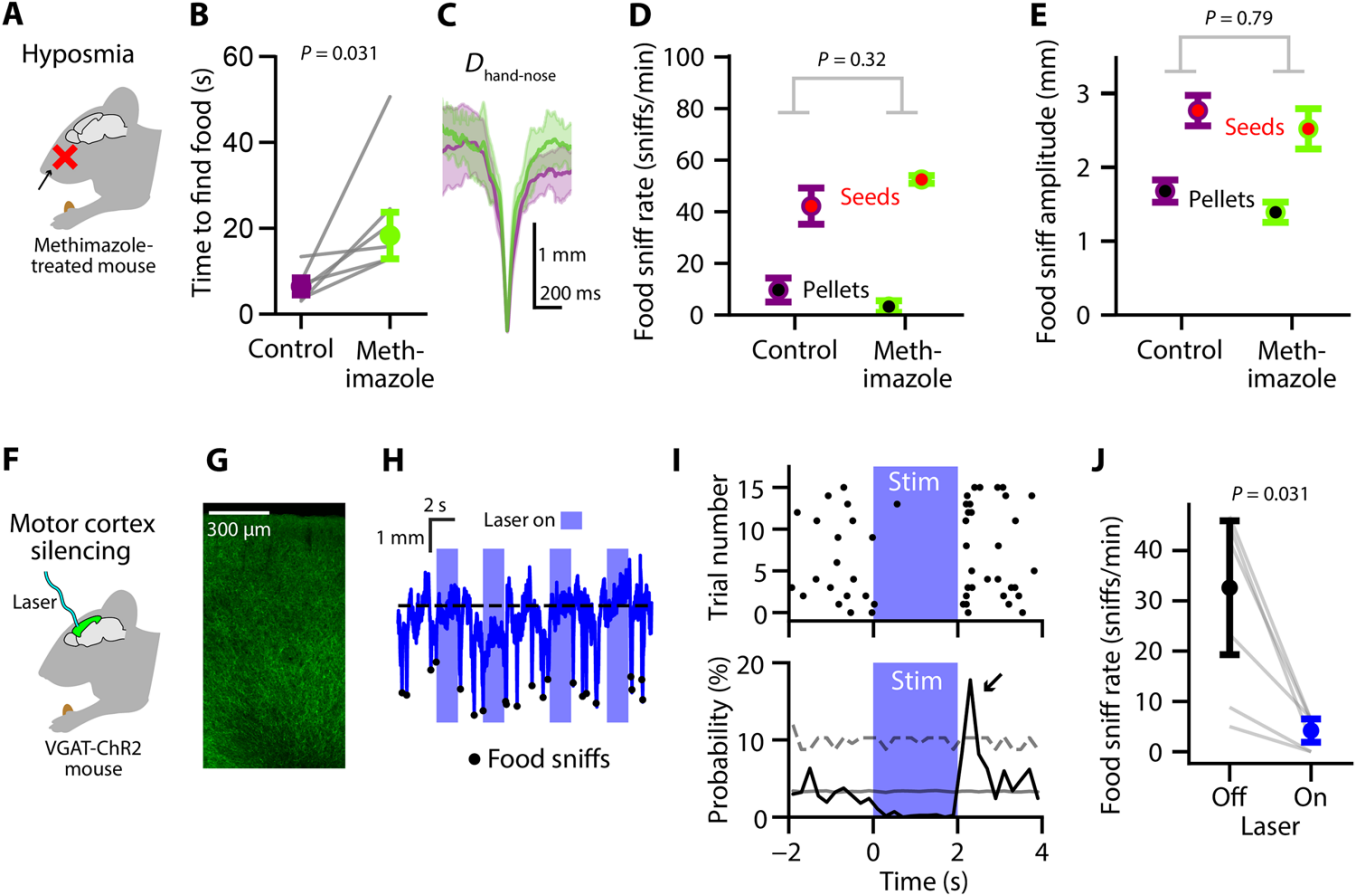
Effects of olfactory and motor manipulations on food sniffs. (**A**) Mice were treated with methimazole to induce hyposmia. (**B**) Estimated time from food delivery to initial food grab, for six mice before (black) and after (red) methimazole. Lines show median values for individual mice. Symbols with error bars show the overall group medians with m.a.d. Recordings were stopped after 60 s (and that value logged) if the mice did not find the food within that time (which occurred sometimes after methimazole; effect size for this group is consequently underestimated). (**C**) Average *D*_hand-nose_ kinematic traces (mean ± SD) for control (purple, six mice) and methimazole-treated mice (green, five mice). (**D**) Comparison of food sniff rates for familiar pellets (black) and novel seeds (red) for control mice (data from Fig. 5B) and methimazole-treated mice (same dataset as fig. S3, day 3). For statistics, see table S2. (**E**) Same, for amplitude. (**F**) Schematic of motor cortex silencing (see text). (**G**) Image shows the labeling pattern in M2 in a coronal slice. (**H**) Example kinematic trace of the hand-nose distance, recorded while delivering blue light stimuli to silence motor cortex activity. (**I**) Top: Raster plot of food sniff events (dots), for multiple sequential trials, aligned to the photostimulus (50 Hz, 5-ms pulses). Bottom: Peristimulus time histogram for food sniff events (black line), averaged across six mice (normalized to sum to one), and plotted with the average of shuffled data (gray solid line) and upper 95% confidence interval (gray dashed line). Note transiently increased food sniffs just after stimulus offset (arrow). (**J**) Food sniff rates (*n* = 6 mice, gray lines) with (black) or without (blue) M2 silencing (median ± m.a.d., Wilcoxon signed-rank test).

The preceding findings indicate that food sniffs exhibit behavioral flexibility and suggest that food sniffs may occur less as a reactive (“sensory-motor”) behavior, externally driven by feeding-associated olfactory stimuli, and more as a proactive (“motor-sensory”) behavior, internally driven by an instinct to intermittently perform a “smell check” on the item that the mouse has decided to consume. To explore this further, in one approach, we treated mice with methimazole, which causes hyposmia by olfactory epithelium ablation (Materials and Methods; Fig. 6A and fig. S4). Methimazole-treated mice had difficulty finding food and took much longer to do so (Wilcoxon signed-rank test, *P* = 0.031, *n* = 6 mice; Fig. 6B and movie S7), confirming treatment efficacy and highlighting the importance of olfaction for obtaining food. However, once food was discovered and grabbed, subsequent food handling behavior, including food sniffs, appeared largely normal (movie S8). Food sniff kinematics were highly similar to controls (Fig. 6C). Also similar to controls, methimazole-treated mice exhibited novelty-related increases in food sniff rate and amplitude, followed by familiarity-related adaptation over ensuing days (Fig. 6, D and E, and fig. S4). Mixed-design ANOVAs showed that the increases in food sniff rate (within factor: food type; table S3) and amplitude (within factor: food type; table S4) were related to food novelty (rate: *P* = 1.4 × 10^−4^, amplitude: *P* = 1.0 × 10^−4^); methimazole treatment had no effect (rate: *P* = 0.32, amplitude: *P* = 0.79), and there was no interaction between novelty and methimazole treatment (rate: *P* = 0.91, amplitude: *P* = 0.75).

Given the lack of effect of hyposmia on food sniffs, we also explored whether an activation-type perturbation, in the form of brief artificial olfactory-like stimuli, might evoke or otherwise affect food sniffs. For this, we performed optogenetic photostimulation of the olfactory bulb in mice previously injected in the olfactory bulb with adeno-associated virus (AAV) carrying channelrhodopsin-2 (ChR2) or green fluorescent protein (GFP) (Materials and Methods; fig. S5). In GFP-injected control mice (*n* = 3), short trains of light pulses did not evoke any responses either during or between feeding bouts. In ChR2-injected mice (*n* = 3), photostimulation was able to evoke a transient increase in breathing rate when mice were not actively handling food, such as between feeding bouts. However, when mice were actively handling food, photostimulation did not evoke food sniffs (fig. S5 and movie S9).

### Motor cortex silencing blocks food sniffs

Complementing the sensory perturbations above, and in light of their lack of effect on food sniff behavior, we also explored the effects of motor cortex silencing on food sniffs to investigate whether they are cortically dependent. To do so, we used VGAT-ChR2 mice, which express ChR2 in cortical interneurons, enabling focal silencing of motor cortex by delivery of blue light to activate these neurons (Fig. 6, F and G, and movie S10). Optical fibers were placed bilaterally in secondary motor cortex (M2), in a region just anterior to forelimb M2 associated with nose movements in prior motor mapping studies (*25*). Mice were first familiarized with food pellets and then given novel food (sunflower seeds) during experiments to promote food sniffs. As shown in an example recording (Fig. 6, H and I), food sniffs appeared to be strongly suppressed when photostimuli (7.5 mW, duration of 2 s) were delivered to bilaterally silence M2 while mice handled food. An increased rate of food sniffs following stimulus offset was also observed (Fig. 6I). Quantification across mice confirmed nearly complete cessation of food sniffs during photostimulation (36.6 ± 13.3 events/min baseline rate, averaged 2 s before and after photostimulation; 4.2 ± 2.3 events/min during photostimulation; median ± m.a.d., *n* = 6 mice, Wilcoxon signed-rank test, *W* = 0, *P* = 0.031) (Fig. 6J). Photostimulation in littermate controls showed no effect (fig. S6). Exploring other cortical areas, we also found no effect of silencing the forelimb region of primary motor cortex (M1) (fig. S6). These results show that food sniffs are suppressed by motor cortex silencing, particularly in M2, and are thus cortically dependent.

## DISCUSSION

By adapting our recording methods to capture subtle millisecond- and millimeter-scale hand-nose-breath interactions below the snout of a feeding mouse, we resolved the clockwork-like sequence of events defining a basic olfactomotor behavior: The mouse intermittently brings food to the nares by movements of the head as well as the hands and, precisely at the closest point, takes a single, quick, well-timed inspiration, in the process of which the breathing rhythm is reset. Together with the effects of behavioral, olfactory, and motor perturbations, these findings establish single food sniffs as a distinct mode of olfactory sampling and carry several implications relating to broader principles of active olfaction and multisystem motor coordination in ethological mammalian behaviors.

First, our findings address a basic question about olfactory behavior: When is a breath primarily an act of olfactory sensing, and when is it primarily for respiratory air exchange? Olfactory sniffing—“voluntary inhalation (or repeated inhalations) in the context of odor-guided behavior”—can be difficult if not impossible to distinguish from nonolfactory breathing (*8*, *9*). The distinction is often made based on a cutoff rate during a sustained bout of faster breathing. In contrast, our findings show that dexterous food sniffs are unitary events, constituting a discrete mode of active olfactory sensing, distinct from the sustained rhythmic forms of sniffing associated with exploration and foraging and from ongoing breathing that meets metabolic needs.

Second, the discreteness of single food sniffs contrasts with the basic tenet that active olfaction is inherently rhythmic (*8*). However, abundant evidence supports the concept of high-speed olfactory processing. Rodents as well as humans can rapidly and precisely control their sniffing to distinguish odors, even novel unlearned odors, on the timescale of an individual sniff (*13*–*16*, *26*, *27*), and sniffs are posited as a fundamental unit of information processing in olfaction (*8*, *24*). Millisecond-scale aspects of olfaction have been shown in operant conditioning studies, including perceptual detection of sniff phase and locking neural activity to olfactory stimuli (*28*–*30*), detection of rapid temporal dynamics in virtual odor movies (*31*), rate discrimination up to 40 Hz (*32*), response times as fast as 60 ms (*33*), and subsniff discrimination of plume odors (*34*). Humans can similarly discriminate two odors presented only 60 ms apart, well under the typical 1- to 3-s duration of single human sniffs (*35*). Our results, demonstrating millisecond-scale time locking of single inspirations with hand and head movements, provide additional evidence for high-speed olfactory processing. Moreover, they show that the capability of single-sniff olfactory behavior, previously established in psychophysics-inspired perception-oriented paradigms as mentioned above, is used naturally in an ethological context, constituting a basic element of food handling, an innate survival-critical behavior. In addition, our findings dovetail neatly with—and provide support for, in a naturally occurring behavior—the hypothesis that sniffing provides the olfactory system with a series of discrete sensory “snapshots” (*8*, *13*). This interpretation thus reconciles single and rhythmic forms of sniffing as discrete and rhythmic variants of a shared underlying process, with individual food sniffs representing the extreme limit of one snapshot (“odor image”). Bolstering this interpretation, food sniffs occasionally occur in brief runs of consecutive events, at frequencies similar to rhythmic olfactory sniffing. The occurrence of food sniffs as single events, rather than extended bouts of sniffing, may reflect a trade-off between obtaining sufficient sensory information for accurate decision making and minimizing consumption time by not having the food under the nares (and thus away from the mouth) longer than necessary.

Third, the abrupt, resetting-like adjustment of the respiratory rhythm observed here contrasts with other forms of resetting. Resetting can be induced experimentally, such as by activating the Breuer-Hering reflex or stimulating respiration-related brainstem nuclei (*21*, *22*), and occurs during sighs, which can be evoked reflexively by hypoxia and other ventilation-related changes (*36*, *37*). Here, we show that an abrupt resetting-like process occurs naturally as a prominent component of a goal-directed behavior. A caveat is that our data are consistent with three possible circuit models. One is that respiratory circuits reset the rhythm and drive the forelimb components of the food sniff. This would be consistent with the idea that, for whisking and other rhythmic orofacial movements, the respiratory rhythm functions as a “master clock” (*17*, *38*, *39*) and further extends it to discrete forelimb movements. A second possibility, hinted at by our cortical silencing experiments, is that forelimb circuits induce a reset of the breathing rhythm. Last, some upstream circuit could drive both the resetting and the forelimb movement. The data do not distinguish between these possibilities, as they make essentially identical predictions about the timing of inspirations around and following food sniffs. Nonetheless, the ability of mice to sniff food at essentially any time point or phase in the ongoing breathing cycle may be adaptive in enabling flexible timing of olfactory sampling during feeding and likely exploits the ability to modulate breathing rate and timing over a wide dynamic range including very short timescales, as discussed above.

Fourth, mice kept on sniffing food as usual despite strong perturbations of the olfactory system causing either the loss of olfactory input (olfactory epithelium ablation) or the delivery of artificial olfactory-like stimuli (olfactory bulb stimulation), perturbations that otherwise caused marked changes in other aspects of foraging and feeding behavior. Perhaps this partly reflects the innateness of food handling behavior, aspects of which suggest the concept of a fixed action pattern (*40*). In addition, a caveat with the optogenetic results is that even though a change in breathing could be triggered, the absence of a specific effect on food sniffs might simply reflect the artificial nature of the stimulus. However, we interpret the persistence of food sniffing behavior despite these perturbations as evidence that food sniffs are primarily a volitional, proactive form of active sensing in the olfactory modality, driven by an impetus to “smell-check” the food in the hands and contrasting with a more reflexive, reactive form in which olfactory stimuli instigate the food sniff. Consistent with this interpretation, transient optogenetic silencing in motor cortex suppressed ongoing food sniffing behavior during food handling. That silencing was effective in M2 may reflect the more premotor-like properties of higher-order motor areas, while the ineffectiveness of M1 silencing accords with previous work showing continued feeding behavior once food is in the hands and being consumed (*5*, *41*), perhaps relating to the distributed encoding of forelimb function (*5*, *42*–*44*). Volitional control of breathing and particularly inspiration is well known in humans (*45*); of relevance to food sniffs, volitional inspiration is associated with enhanced sensory sampling and cortical excitability (*46*, *47*). Neuroanatomical pathways mediating such control are complex, involving corticobulbar and corticospinal projections from motor cortex that feed into premotor and pre-premotor circuits controlling breathing in coordination with other behaviors (*45*). Delineating those that drive single food sniffs is a future priority.

The multifaceted features of single food sniffs present many signposts for further study of this complex behavioral motif. For example, one line of inquiry concerning the motor aspects is to identify the circuit-level mechanisms mediating the tight yet transient coordination across multiple systems. A second, concerning the sensory aspects, is to explore the roles of olfactory perception and decision making, such as by systematically varying stimulus properties such as odor type and intensity. A third, from an ecological-ethological perspective, is to understand how optimization of foraging strategies, energetics, and related cost/benefit trade-offs factor into the behavior.

For humans, the act of bringing food to one’s nose and sampling its aroma with a single sniff—a discrete, deliberate inhalation—prior to ingestion is a commonplace yet motorically complex feat of multisystem dexterity that coordinates oromanual manipulation and active olfaction. For other mammals, rhythmic rather than discrete forms of sniffing have been considered the natural mode of active olfaction, as when tracking scent trails and odor plumes. However, as discussed above, evidence from psychophysical paradigms in rodents as well as humans indicates a capacity for rapid olfactory processing, on the timescale of single sniffs or even faster, but whether and how rodents may naturally exploit these high-speed olfactory capabilities have been unclear. Our results establish single food sniffs as a mode of discrete, high-speed active olfaction in mice. The findings point to an unexpected parallel between active olfactory behavior in humans and rodents, and raise the possibility of single-sniff olfaction in other mammals as well. This could include mammals lacking manual dexterity, as the large contribution of head movements to the kinematics implies that “cephalic dexterity” alone should suffice for single-sniff olfactory sampling. As an extreme example, mammals with agile elongated noses (Macroscelididae and Proboscidae) appear to bring the nose to the odor source, rather than vice versa, but we speculate that single-sniffing should be possible and even common among any mammals capable of rapid directional nose or head movements.

## MATERIALS AND METHODS

### Animals

Animal studies were approved by Northwestern University Institutional Animal Care and Use Committee (IS00025618) and fully complied with the animal welfare guidelines of the National Institutes of Health and Society for Neuroscience. There were 62 mice used in this study (table S5). For most experiments, wild-type mice (strain C57BL/6; stock no. 000664, The Jackson Laboratory) were used, in an age range of 9 to 24 postnatal weeks and weighing at least 20.0 g. A subset of experiments used VGAT-ChR2 mice [B6.Cg-Tg(Slc32a1-COP4*H134R/EYFP)8Gfng/J; stock no. 014548, The Jackson Laboratory] (*48*). Both male and female mice were used. Mice were kept on a 12:12-hour reverse light/dark cycle, with behavioral testing occurring during the dark phase of the cycle.

### General surgical procedures

For all surgeries, deep anesthesia was induced and maintained with either inhalational isoflurane (3 to 5% for induction, 1 to 2% for maintenance) or ketamine (10 mg/kg)/xylazine (1 mg/kg) cocktail administered intraperitoneally. The body temperature was maintained at 37°C with a heating pad. Ophthalmic ointment was applied to the eyes. Buprenorphine (1 mg/kg) was administered subcutaneously prior to surgery. Bupivacaine (<2 mg/kg) was administered subcutaneously at the site of incision before surgery to provide local anesthesia. The surgical area on animals was wiped with 70% ethanol and betadine alternately three times to sterilize. Meloxicam (20 mg/kg) was administered subcutaneously after surgery every 24 hours for up to 72 hours.

### Thermistor implantation

To monitor breathing, a thermistor (GAG22K7MCD419, TE Connectivity Measurement Specialties) was implanted in one side of the nasal cavity above the olfactory epithelium as previously described (*20*). An opening was made with a microdrill on the nasal bone 0.5 mm lateral from the midline suture and 3.1 mm from the nasal fissure. The site to implant was stereotaxically targeted. The distal half of the thermistor was inserted into the cavity between the nasal bone and olfactory epithelium. The rest of the thermistor was covered by Kwik-Sil (World Precision Instruments) to protect it from dental cement. The thermistor and a custom-made connector (CLP-112-02-F-D-A, Samtec) were then fixed on the cranium and the nasal bone with dental cement (C&B Metabond, Parkell).

### Virus injection and optical fiber implantation

In a subset of experiments, we injected the olfactory bulbs with AAV carrying channelrhodopsin [pAAV-CaMKIIa-hChR2(H134R)-EYFP, 26969-AAV1, Addgene] or GFP [pAAV-CAG-GFP, 37825-AAV1, Addgene] for controls. General surgical procedures were as described as above. A craniotomy was opened to expose one olfactory bulb (in millimeter: anteroposterior 3.8, mediolateral ±0.5), and 200 nl of virus (titer ≥ 1 × 10^13^ vg/ml) was injected using a Nanoliter 2020 Injector (World Precision Instruments) at 0.5 nl/s at a depth of 300 μm. During thermistor implantation (described above), an optical fiber cannula (ceramic ferrule, 0.39 numerical aperture, Ø200-μm core, 0.5-mm exposed fiber, custom made by RWD Life Science) was implanted over the injection site. The cannula was fixed with the dental cement on the cranium. For motor cortex silencing, bilateral craniotomies were made over secondary motor cortex (in millimeter: anteroposterior 2.5, mediolateral ±0.75 to 1.25) or forelimb primary motor cortex (anteroposterior 0.2, mediolateral ±1.5), followed by implantation of the optical fiber cannulae.

### Methimazole treatment

In one set of experiments, we treated mice with methimazole, a chemical treatment that ablates the olfactory epithelium and causes severe hyposmia (*49*–*53*). Methimazole (75 mg/kg; Vetranal, 46429, Sigma-Aldrich) was injected intraperitoneally. Mice were allowed to recover for a day before further experiments.

We confirmed the efficacy of methimazole for inducing hyposmia using an olfactory cross-habituation assay (fig. S4) (*54*). Isoamyl acetate, limonene, and 2-heptanone were diluted 1:1000 in mineral oil. Animals were tested in their home cages, with new clean bedding provided 24 to 40 hours before testing. Each odorant was applied to the swab of a cotton-tipped applicator stick. The swab was then covered by odorless plastic tubing to prevent mice from contacting it. Odorants were presented to mice by inserting the stick through a port on the side of the cage. Each odorant was presented over four successive trials lasting 20 s each, separated by 30-s intertrial intervals. Video recordings were analyzed offline to determine how much time mice spent investigating the odorant, defined as snout-oriented investigation within 1 cm of the odor stick. The cohort comprised 12 wild-type mice divided into two groups of six. One group was treated with methimazole (75 mg/kg, intraperitoneal injection) 48 hours before the test; the control group was treated with saline. The experimenter was blind to the methimazole treatment.

### Multimodal behavioral recording chamber

To record video with multiple camera views along with breathing signals and to deliver food and optogenetic stimuli in freely moving mice, we designed and constructed a multimodal behavioral recording chamber. The main body of the chamber was built with 3D-printed plastic frames and transparent polycarbonate (8707K129, McMaster-Carr) walls and floor. The chamber was elevated on posts so we could record behavior videos from multiple angles including from below.

One high-speed camera (Chameleon3 USB3 CM3-U3-13Y3M-CS, Teledyne FLIR), equipped with a zoom lens (A4Z2812CS-MPIR, Computar, with an additional zoom in singlet lens), was mounted below the chamber facing upward to provide a zoomed-in ventral (bottom) view of the mouse through the clear chamber floor. This camera was mounted on an *x*-*y* pair of linear actuators, driven by stepper motors (V-Slot NEMA 17 Linear Actuator Bundle, OpenBuilds Part Store, Zephyrhills, Florida) that were controlled by a microcontroller (Arduino Uno, Arduino) and motor drivers (EasyDriver, SparkFun). A top-view camera (Raspberry Pi Camera v2 NoIR) was mounted above the chamber to track the animal’s location. The top-view camera’s video output was acquired by a Jetson Nano developer board (Nvidia). The locations of the ears of the animal were tracked from the video stream in real time with DeepLabCut-Live (*18*) using pretrained neural networks. The top-left and bottom-right corners of the arena were also detected at the start of each session and used to define the arena area. A camera target coordinate was calculated using the location of the two ears and the corners. The arena floor was divided into a 100 × 100 grid virtually, and the camera target location was transferred onto these grids as *x*-*y* coordinates and sent to the microcontroller through USB communication. Four limit switches were placed on the two bottom-view camera tracks to set the moving range of the camera, such that the center of the camera view could just reach the four corners of the arena before triggering the switch. During initialization of the motor control system, the camera was moved to reach and trigger all limit switches to detect and log the physical dimensions of the desired moving range. The camera target coordinate (100 × 100) commands from the Jetson Nano were translated into distance, and the steps needed to move in both axes were calculated.

Additional cameras (CM3-U3-13Y3M-CS or BFS-PGE-04S2M-CS Teledyne FLIR) were mounted on the sides of the chamber. These were equipped with lenses (1/3-inch, 2.8 to 12 mm, *f*/1.4, Vari-Focal Lens with Connector, Tamron) and positioned to view the chamber through a prism and pair of mirrors to provide paired side-view images in each camera’s video recording, as previously described (*5*). This two-camera system thus yielded four simultaneous views of the mouse, from different angles. These stationary cameras had a larger field of view than the robotic bottom-mounted camera.

### Behavioral testing

During an initial habituation period, mice were placed in the behavior chamber for sessions of 90 min/day over at least 2 days. During the food handling behavioral session, small round food pellets (F0165, Dustless Precision Pellets, 45 mg, rodent grain-based diet, Bio-Serv) were dropped from the top of the chamber at least 5 min after the mice were placed in the chamber. One food pellet was delivered each time, and the intervals of the delivery were >80 s. Delivery continued until food was not picked up within 5 min, in which case the session was terminated. Mice were given 10 to 21 pellets on a single day.

In a subset of experiments, delivery of food pellets was alternated with sunflower seeds (S5137-1 sunflower seeds, black oil, gamma-irradiated, Bio-Serv). The sunflower seeds were hulled and cut in half before delivery to the mice, to make the pieces more comparable to the pellets (pellets: 45 ± 2 mg, ⌀ 4.0 ± 0.1 mm; half-seeds: 29 ± 4 mg, ⌀ 4.3 ± 0.5 mm; mean ± SD, *n* = 30 items of each type).

In another subset of experiments, we crafted food-coated fecal balls using each mouse’s own fecal droppings (chopped into small pieces), distilled water, and ground-up food pellets. “Regular” food balls were made in the same way but without feces cores. The two types of food balls were delivered to mice in an alternating sequence.

All mice were food-restricted to 85 to 90% body weight for behavior testing to improve the efficiency and shorten the experimental sessions. Mice were fed with their usual rodent grain-based diet (7912 Teklad LM-485 mouse/rat sterilizable diet, Inotiv) but restricted to a single measured amount per day to maintain body weight within the target range. Food restriction began at least 3 days after surgery and at least 2 days before the habituation.

### Optogenetic photostimulation

A blue laser (MDL-III-470/100 mW, Opto Engine) was used as the light source. A rotary joint splitter (RJ2, Thorlabs) was used to connect the optical fiber cable (FT200EMT) from the laser to the animal. The laser (7.5 mW at ⌀ 0.2 mm fiber tip) was controlled through a transistor–transistor logic (TTL) trigger signal sent by a microcontroller (Arduino Uno, Arduino). The TTL signal was recorded by a data acquisition board (NI USB 6229, National Instruments) together with other signals. Light pulses (5 ms, 50 Hz, 25% duty cycle) were used at various durations.

### Data acquisition and analysis: Kinematics

Video recordings were made with a high-speed camera at 300 fps, 2.79- to 2.90-ms exposure with 850-nm infrared lighting from light-emitting diode (LED) strips (7031.85, Waveform Lighting). The acquisition of all frames was triggered by a TTL signal, and the exposure output TTL was recorded by a DAQ board (NI USB 6229, National Instruments) to ensure synchronization.

DeepLabCut (*19*) was used to track the nose tip, nares, philtrum, the proximal and distal interphalangeal joints on the second to fifth digits of the hands from the bottom-view videos. The 1923 labeled frames were used to train a ResNet-101 neural network for 560,000 iterations. The average hand position was defined by the midpoint between the two hands of the tracked proximal interphalangeal joints on the D4 and D5. The *D*_hand-nose_ was defined as the distance between the nose and the average hand position. The running mean with a 2-s window was subtracted from the *D*_hand-nose_ to generate a baseline-subtracted *D*_hand-nose_. The time point where the running mean-subtracted *D*_hand-nose_ exceeded a threshold of 13 pixels (~0.7 mm) was used to initiate the search for local minima. The minima were searched in a 40-ms time window after the initiation points, and the newly found minima were used for the left edge of the window for the next iteration of the search. The search stopped when the difference between the newly found minimum and the previous one was less than two pixels (~0.1 mm). The sniff movement amplitude was calculated by subtracting the median during food holding from the *D*_hand-nose_ minimum. Events with an amplitude of less than 15 pixels were excluded. The tracked points with prediction confidence (confidence given by the trained neural network) less than 0.99 were excluded from the analysis.

To analyze the head and hand contributions to the kinematics, we used the philtrum of the mouse to define the longitudinal axis and determined the separate trajectories of the head and hands along this axis. For each sniff event, we used the peri-sniff traces (±250 ms around the minimum-*D*_hand-nose_) to find the maximum (head) or minimum (hands) of the running average with a 30-ms time window in the head and hand traces, respectively, and used these to baseline-subtract the traces.

Epochs of rearing or foraging—when the animal either assumes a stationary bipedal stance with its head up to “sniff the air” or uses quadrupedal locomotion with its head down close to the substrate while searching for food (*55*)—were identified by visual inspection of the side-view videos. Although movements of other body parts were also evident in the movies, such as whisker movements during foraging-related epochs, these were not quantified as our imaging system was not optimized for tracking them. Similarly, our recordings do not address or exclude the possibility that somatosensation (e.g., involving smaller submental and other perioral vibrissae) may additionally be involved in single food sniffs.

### Data acquisition and analysis: Breathing signals

The implanted thermistor was balanced with a Wheatstone bridge to remove the dc shift before the recording started. The voltage changes were recorded and digitized with a custom-built amplifier and a DAQ board (NI USB 6229, National instrument) at a 10- or 50-kHz sampling rate.

The thermistor signal was separately filtered with a fourth-order 30-Hz low-pass Butterworth filter and a fifth-order 100-Hz low-pass Butterworth filter to generate two low-pass filtered signals for all thermistor recordings. The local extrema were found on the 30-Hz low-pass filtered signal and then used to initiate a ±10-ms search window for the local extrema on the 100-Hz low-pass filtered signal. The extrema were found by running a comparator (greater or less) along the signal section in the search window and taking the first points that returned False. The extrema that satisfied the following criteria were considered as start points of inspiration or expiration: (i) The inspiration or expiration duration was between 14 and 500 ms; (ii) the amplitude of the signal was greater than 0.5 mV.

To analyze the instantaneous phase of the breathing signal, the thermistor signal was 1- to 30-Hz bandpass-filtered to compute the phase value of the breathing signal over time. The analytic signal *Z*(*t*) and instantaneous phase ϕ(t) were computed with the Hilbert transform of the filtered signal S(t) as

Z(t)=S(t)+jH[S(t)]

ϕ(t)=arg[Z(t)]

where H[S(t)] is Hilbert transform of the filtered signal S(t), and arg[Z(t)] was the argument of the analytic signal *Z*(*t*). A 2D phase-time histogram for each animal was computed using 150 temporal bins (~6.7 ms) over 1-s recording and 36 phase angle bins (10°). The average distribution of the breathing phase was calculated by averaging the phase-time histograms of all animals. PLVs were calculated separately for the 2D phase-time histograms for each animal and for the average phase-time histogram. PLV was defined as (*56*, *57*)

$$\begin{array}{c} \text{PLV}=|E\left[e^{j\Delta \phi \left(t\right)}\right]|\\ =\frac{1}{M}\sum_{m=1}^{M}f_{\phi_{m}}\left(t\right)e^{j\Delta \phi_{m}\left(t\right)} \end{array}$$

where E is the expectation, *m* is the phase bins, and $f_{\phi_{m}}\left(t\right)$ is the frequency of phase $\phi_{m}$ at time *t*. The Δϕ(t) was the phase at time *t* relative to the start point of inspiration (−π). The bias of the PLV estimator was then corrected using the following method (*58*)

$$\gamma_{n}=\frac{1}{2}\sqrt{\frac{\pi }{n}}$$

$${\text{PLV}}_{\text{unbiased}}=\frac{\text{PLV}-\gamma_{n}}{1-\gamma_{n}}$$

where *n* is the sample size.

A similar analysis was performed using randomly chosen non–food-sniff inspirations that occurred close in time to food-sniff inspirations. Specifically, for each food sniff event, we chose a non–food-sniff inspiration within ±0.5 s of the food-sniff inspiration. We then shifted the trace (i.e., all the inspiration timings) by the time difference between the non–food-sniff inspiration and the food-sniff inspiration. This approach resulted in recentering of the trace on the non–food-sniff inspiration, while also preserving the original time difference (Δ*t*) between the *D*_hand-nose_ minimum and the food-sniff inspiration for each food sniff event. This random process was repeated for 10,000 iterations for the statistical comparison.

To analyze phase resetting of the breathing rhythm, we adapted the method of Paydarfar *et al.* (*21*, *22*) as follows. We used the kinematically defined food sniff (instead of an experimentally applied stimulus) to define the event timing for the analysis. We used the inspiration that was closest in time to the food sniff event to define the zeroth inspiration in the peri-sniff breathing cycles. We allowed phases to be negative, to allow for the possibility that the zeroth inspiration could precede the kinematically defined event. We measured the time from the inspiration preceding the zeroth inspiration to the kinematic peak (defined as the time that *D*_hand-nose_ reached minimum), and the cophase (θ) as the time from the kinematic peak to six of the peri–food-sniff inspirations (preceding inspirations, food-sniff inspiration, and two succeeding inspirations). By this definition, the phase of inspiration preceding the zeroth inspiration is the additive inverse of the cophase. These times were normalized by dividing by the median breathing cycle duration in each video, thereby obtaining the normalized phase and cophase. This normalization procedure sometimes resulted in phase values greater than one.

We considered three hypotheses for how food sniffs might relate to the ongoing breathing rhythm. Hypothesis 1 (nonindependence): Food sniffs might be statistically dependent on the ongoing breathing rhythm, in which case the slope of the phase/cophase plot (Δθ/Δφ) will depend on the correlation between subsequent breathing cycle durations. In the extreme case that subsequent breathing cycle durations are statistically independent, this will give Δθ/Δφ = 0, similar to the resetting model. Hypothesis 2 (nonresetting): Food sniffs are independent of the breathing rhythm and there is no resetting. Hypothesis 3 (resetting): The breathing rhythm resets around food sniffs. In the case of a steady, low-variance breathing rhythm, hypotheses 2 and 3 predict slopes of −1 and 0, respectively, as depicted in Fig. 2F. However, in practice, the breathing rhythm varies from cycle to cycle and over time. To empirically determine the expected values of the slope and the coefficient of determination (*R*^2^) of the regression of cophase on phase for each cycle under each of these hypotheses, we ran three different shuffled controls (fig. S3A). For hypothesis 1, we took each kinematic trough and reassigned it to a different, non–food-sniff inspiration, preserving the relative timing. For hypothesis 2, we uniformly randomly inserted a sham event between two non–food sniffs and aligned to these (unlike all other shuffled controls, this forces the cophase of cycle zero to be zero). Last, for hypothesis 3, we took each kinematic trough and reassigned it to a randomly chosen non–food-sniff inspiration (as for the nonindependent model) and then moved the following inspiration so that its cophase matched that of the first non–food-sniff inspiration following a different randomly chosen food sniff. To understand why this simulates resetting, consider the definition of type 0 resetting from Paydarfar *et al.* (*21*), i.e., no net change in cophase as phase varies from zero to one. Shuffling the data in this way amounts to randomly drawing a new cophase for the inspiration after the reassigned food sniff from the real distribution of kinematic trough to subsequent non–food-sniff inspiration intervals, independent of the old phase. By construction, this gives a flat phase/cophase plot (slope ≈ 0), so the net change in cophase as phase goes from zero to one is zero, i.e., type 0 resetting. Example phase/cophase plots under each of these models are shown in fig. S3B. Each model was run for 1000 iterations for each mouse, and medians were taken over mice for each iteration to estimate the distribution of regression parameters for each cycle under each model (fig. S3C). These distributions were used to assign a *P* value to the likelihood of observing the real slope and *R*^2^ under each model, with low values taken to indicate that a given model does not adequately explain the observed data, and large values that a given model cannot be ruled out on the basis of available data.

### Analyses of timing and phase locking

To test whether food sniff kinematics and inspiration were temporally correlated, randomly generated food sniff events were used to compare experimentally observed food sniff events. For each observed food sniff event, a sham food sniff event was generated by randomly assigning a time from a uniform distribution in the range of the breathing cycle where the observed food sniff happened. The Δ*t* between the sham food sniff and the closest inspiration was calculated. The median and the m.a.d. were then calculated from the Δ*t* values. This process was repeated one million times to generate the distributions of the m.a.d., and compared to the m.a.d. of the observed Δ*t* values.

To test whether the PLV was significantly higher around the food sniffs, the sniff event times for each sniff event were randomly shifted in time by 0 to 1 s, and this was repeated 10,000 times. The PLVs of shuffled data were used to compute a confidence interval and compared to the PLVs of the observed events.

### Statistical analyses

Descriptive statistics of data are reported as the median ± m.a.d. or mean ± SD as indicated. Group comparisons were made nonparametric tests whenever possible, with significance defined as *P* < 0.05. Signed-rank tests were used for paired data unless otherwise indicated; e.g., paired *t* tests in the case of smaller sample sizes (*n* < 6). Mixed-design or repeated measures ANOVA were used to compare multiple groups as appropriate. Bonferroni correction was used for multiple comparisons. The two-sample KS test was used to compare distributions.
