# Supplementary material for: Dexterous single sniffs for ethological active olfaction
Source: Sci Adv. 2026 Jul 3;12(27):eaed3610. doi: 10.1126/sciadv.aed3610 (PMC13339859; doi:10.1126/sciadv.aed3610)
Supplement: Supplementary file 1 — Figs. S1 to S6 Tables S1 to S5 Legends for movies S1 to S10 [file sciadv.aed3610_sm.pdf]

Science Advances
AAAS

Supplementary Materials for

Dexterous single sniffs for ethological active olfaction

Mang Gao *et al.*

Corresponding author: Mang Gao, mang.gao@northwestern.edu; John M. Barrett, john.barrett@northwestern.edu; Gordon M. G. Shepherd, g-shepherd@northwestern.edu

Sci. Adv. **12**, eaed3610 (2026)
DOI: 10.1126/sciadv.aed3610

The PDF file includes:

Figs. S1 to S6
Tables S1 to S5
Legends for movies S1 to S10

Other Supplementary Material for this manuscript includes the following:

Movies S1 to S10

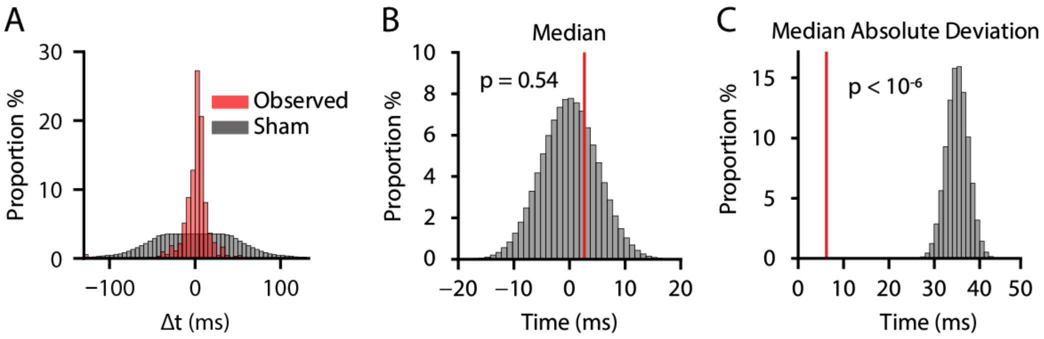

Fig. S1. Statistical analysis of food sniff timing.
(**A**) The overall average histogram of the observed time differences (Δt) between the extrema of the kinematic and breathing data during the food sniffs (time of minimum-$D_{hand\text{-}nose}$ minus the start time of the closest inspiration)(red) is plotted together with that for sham events (gray), generated by randomizing the Δt of the kinematics and breathing over 10^6 iterations. Note that the sham distribution is a uniform distribution with exponential-like flanks, which reflect the variability of breathing cycle durations.
(**B**) The distribution of the medians for each of the 10^6 iterations of randomly generated Δt values (gray) is plotted along with the median of the average distribution of the observed events (red vertical line, same as that plotted in **Fig. 1K**, bottom plot, right inset, "Median"). The medians of the sham Δt values were distributed around 0 ms, and the median of the observed Δt values (2.7 ms) was not significantly different from the sham events ($p = 0.54$, based on the shuffled distribution)
(**C**) Same as panel B, for the m.a.d. of the sham data (gray) and observed data (red vertical line, same as that plotted in **Fig. 1K**, bottom plot, right inset, "m.a.d."). The distribution of the sham events (m.a.d. = 28.9 ± 1.5 ms, mean ± s.d.) was much wider than that of the observed events (m.a.d. = 6.2 ms, $p < 10^{-6}$, based on the sham distribution).

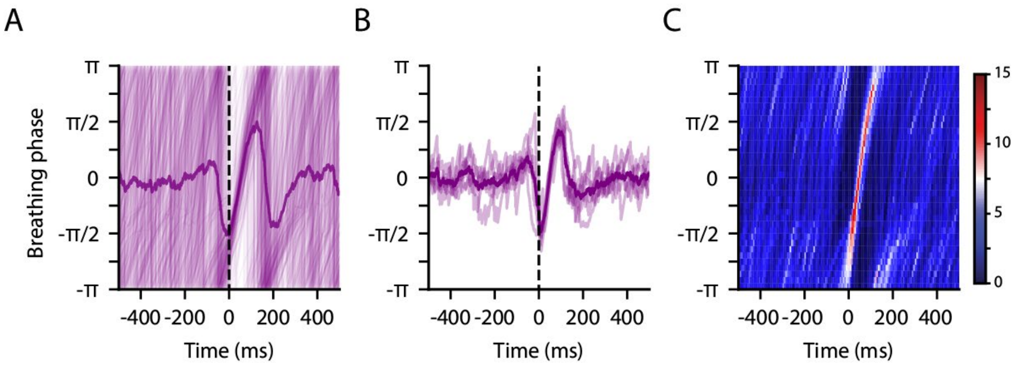

Fig. S2. Analysis of phase-locking using non-food-sniff events.
(**A**) Example recording from one mouse of the breathing phase during non-food sniff events. Traces for individual events (thin lines) were aligned to the time the $D_{hand\text{-}nose}$ reached the minimum (at time = 0 ms), and plotted along with the average across food sniff events (thick line). The inspiration start phase was set to -π.
(**B**) Average breathing phase of individual mice (thin lines) and the grand average across mice (thick lines).
(**C**) Average 2D distribution of the breathing signal phase over time, for the observed breathing signal data. The time $D_{hand\text{-}nose}$ reached minimum was used as time zero. The histogram is normalized along phase (the sum in each time bin equals 1).

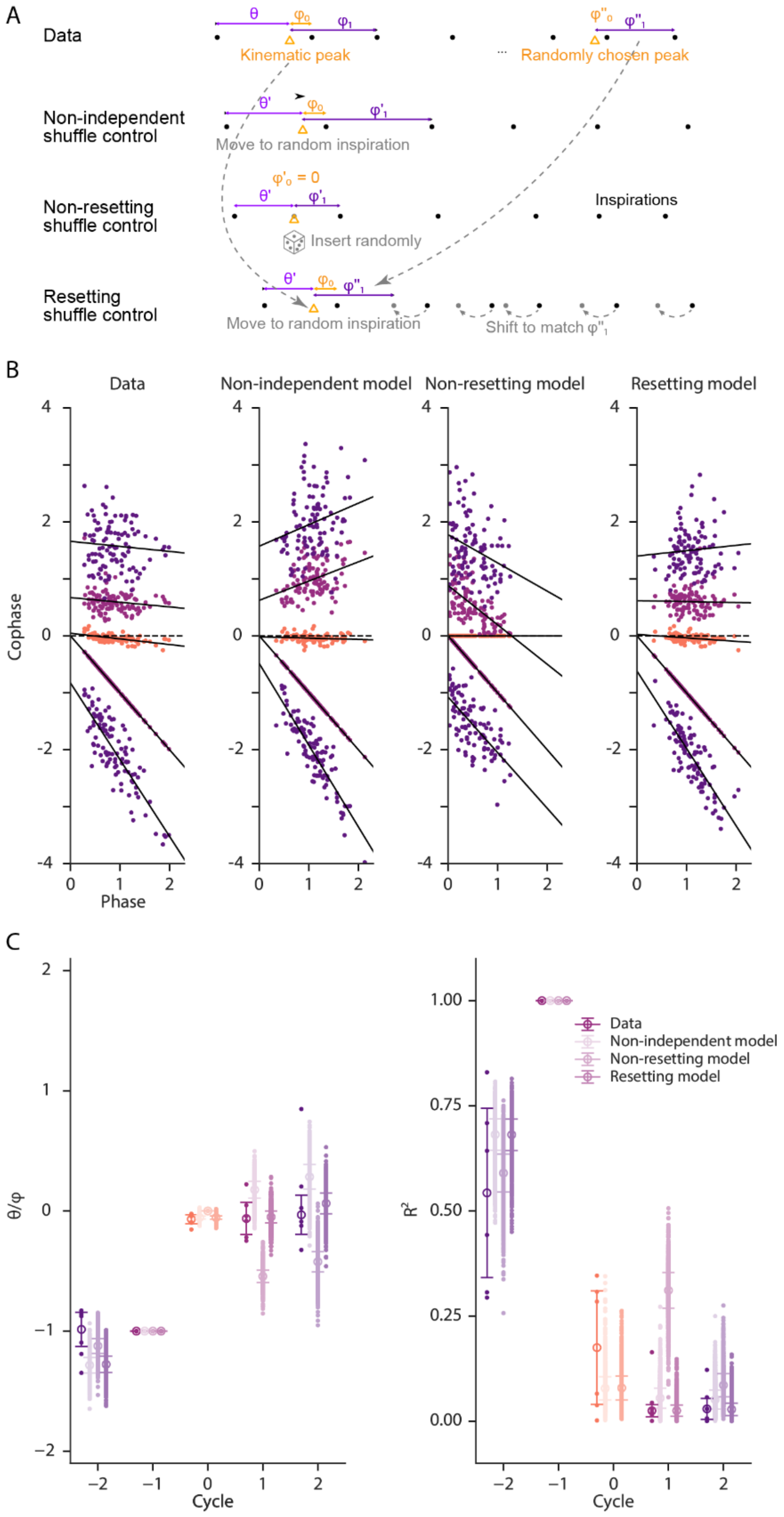
A
Data
θ
φ0
φ1
Kinematic peak
φ"0
φ"1
Randomly chosen peak
Non-independent shuffle control
θ'
φ0
φ'1
Move to random inspiration
Non-resetting shuffle control
φ'0 = 0
θ'
φ'1
Inspirations
Insert randomly
Resetting shuffle control
θ'
φ0
φ"1
Move to random inspiration
Shift to match φ"1
B
Data
Non-independent model
Non-resetting model
Resetting model
Cophase
Phase
C
θ/φ
Cycle
R²
Cycle
Data
Non-independent model
Non-resetting model
Resetting model

Fig. S3. Analysis of resetting using shuffled data.
(**A**) Schematic of shuffled controls to determine the distributions of phase/cophase regression parameters under different hypotheses about inspiration timing relative to food sniff kinematics.
(**B**) Example phase/co-phase plots taken from the real data (far left, the same as **Fig. 2G**) and under the non-independent (middle left), non-resetting (middle right), and resetting models (far right).
(**C**) Coefficient of determination (R^2) of the phase/co-phase regression lines for each cycle for the real data (darkest symbols, dots are mice, error bars are median ± m.a.d. over mice) and over 1000 iterations of each model (from palest to darkest: non-independent, non-resetting, resetting; dots are medians over mice per iteration, error bars are medians over mice and iterations ± m.a.d. over iterations of the median over mice).
(**D**) Same, for slope.

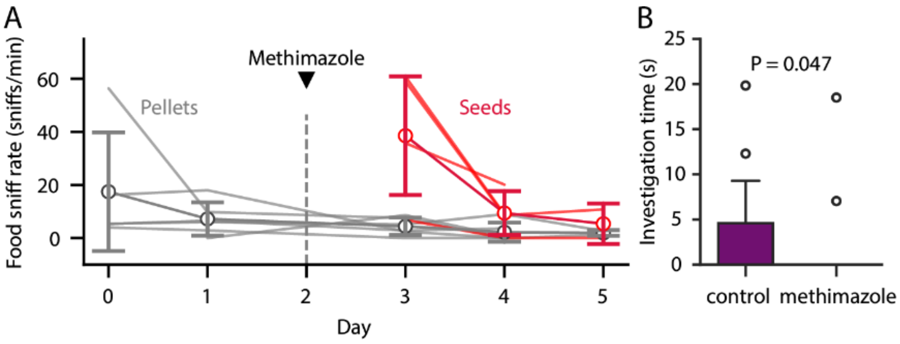

Fig. S4. Additional analyses of methimazole treatment.
(A) Food sniff rate (number of food sniffs per minute) versus day of behavioral session, for a cohort of mice (n = 6) treated with methimazole on Day 2, feeding on food pellets (gray; first given at Day 0) and sunflower seeds (red; first given at Day 3). Data are plotted as the mean ± s.d. Data on some days have fewer data points for sunflower seeds, due to some methimazole-treated mice not feeding on them on those days.
(B) Cross-habituation assay to assess effect of methimazole treatment on olfactory behavior (see **Methods**). The plot shows the average amount of time that control mice (n = 6) and methimazole-treated mice (n = 6) spent investigating novel odors, for three different odors presented through a port in the side of the home cage. The boxplots show the quartiles and 1.5 times the interquartile ranges (IQR) of the investigation times, pooled over odors for each group of mice. Outliers are determined as the data points exceeding 1.5 times IQR above the third quartile. Mann–Whitney U test, U=112.0, $n_1 = n_2 = 18$.

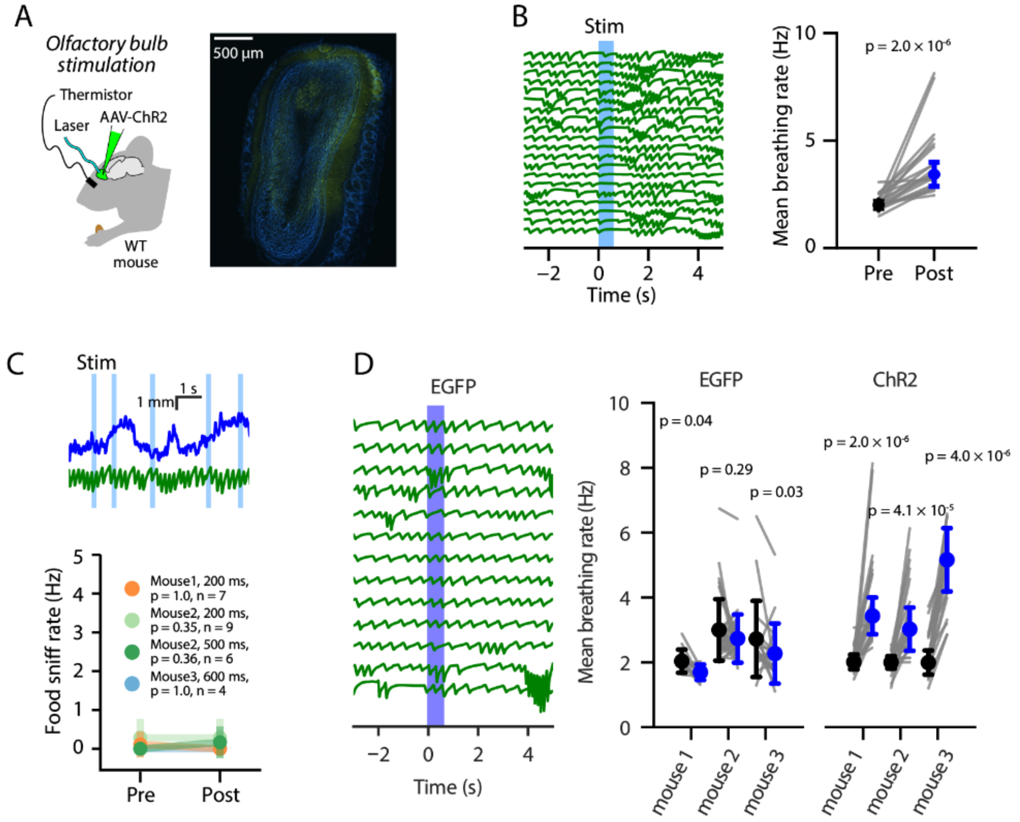

Fig. S5. Optogenetic olfactory bulb stimulation.
(**A**) Left: Schematic of the labeling and recording paradigm. Right: Fluorescence image of ChR2-EYFP expression in olfactory bulb.
(**B**) Left: Example breathing traces, recorded during olfactory bulb stimulation (Stim), while the mouse was resting. Right: Average breathing rate measured 3 breathing cycles before (Pre, black) or after (Post, blue) stimulation (median ± m.a.d., W = 0, n = 20 trials).
(**C**) Left: Example kinematic (blue) and breathing signal (green) traces recorded during olfactory bulb stimulation (vertical blue bars), while the mouse was handling food. Right: Average food sniff rate 1 s before versus 1 s after the stimulus. Paired t-test was used to test significance, sample size n indicates numbers of trials.
(**D**) EGFP control for olfactory bulb stimulation experiment. Left: Example traces are from one mouse injected with EGFP in the olfactory bulbs, showing lack of effect of blue light stimulation on the breathing rhythm. Right: Average breathing rate of mice before (black) or after (blue) stimulation (median ± m.a.d.) for ChR2 expressing mice or EGFP controls (Wilcoxon signed-rank test; Control mice: W = 13.0, n = 12 trials; W = 281.0, n = 37, W = 47.0, n = 20. Chr2 mice: W = 0, n = 20; W = 10.0, n = 21; W = 0, n = 19).

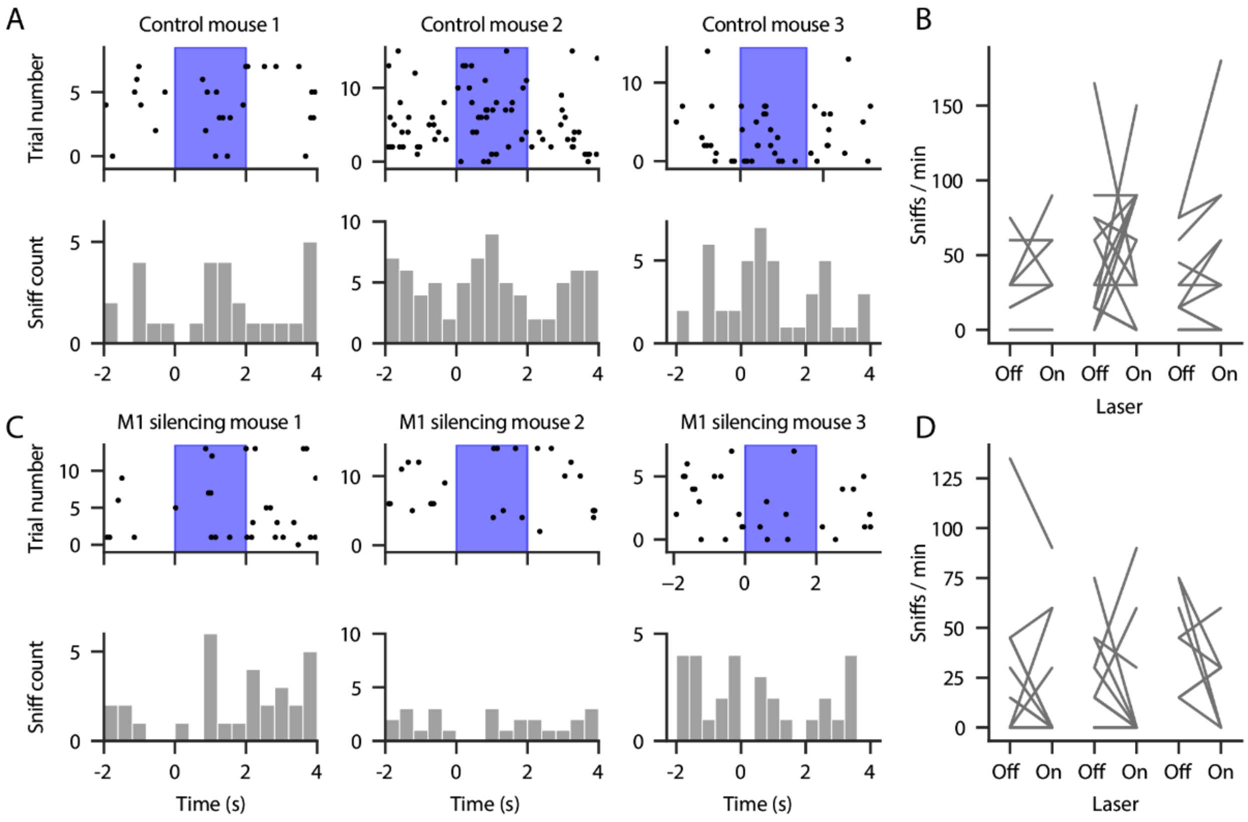

Fig. S6. Photostimulation in M2 in ChR2-negative littermate control mice, or in forelimb M1 in ChR2+ mice, did not affect food sniffing.
(A) Top: Raster plot of food sniff events (dots) in control (VGAT-ChR2 negative littermate) mice, for multiple sequential trials of photostimulus presentation, aligned to the photostimulus (50 Hz, 5 ms pulses). Bottom: Peristimulus time histogram for food sniff events (gray).
(B) Food sniff rates of the three mice, Wilcoxon signed-rank test, W = 4, p = 0.38, n = 9 trials (mouse 1); W = 37.5, p = 0.34, n = 16 trials (mouse 2); W = 9, p = 0.10, n =15 trials (mouse 3).
(C) Same as (A), for a VGAT-ChR2 positive mice in which cortical silencing was targeted to forelimb M1 bilaterally.
(D) Median food sniff rates of the two M1 silencing mice, Wilcoxon signed-rank test, W = 15.5, p = 0.40, n = 14 trials (mouse 1); W = 14.5, p = 0.34, n = 15 trials (mouse 2); W = 9, p = 0.266, n = 8 trials (mouse 3).

Supplementary Tables

Table S1. Repeated measures ANOVA for effect of novelty and food type on food sniff rate.

Source	Sum of squares	Degrees of freedom (numerator)	Degrees of freedom (denominator)	F-values	p-values	Partial eta-squared effect sizes	Sphericity
Novel	3545.55	1	5	19.76	0.01	0.52	TRUE
Food type	1857.54	1	5	12.34	0.02	0.36	TRUE
Novel * Food type	1160.99	1	5	8.46	0.03	0.26	TRUE

Table S2. Pair-wise comparison for effect of novelty and food type on food sniff rate.

Contrast	Novel	A	B	Paired	W-values	Alternative	p-uncorrected	p-corrected	p-adjust method	Hedges
Novel	-	FALSE	TRUE	FALSE	0	two-sided	0.03	N/A	N/A	-2.37
Food type	-	Pellets	Seeds	FALSE	0	two-sided	0.03	N/A	N/A	-1.79
Novel * Food type	FALSE	Pellets	Seeds	FALSE	4	two-sided	0.22	0.22	Bonferroni	-0.81
Novel * Food_type	TRUE	Pellets	Seeds	FALSE	0	two-sided	0.03	0.06	Bonferroni	-1.66
Food type * Novel	Pellets	FALSE	TRUE	TRUE	0	two-sided	0.03	0.06	Bonferroni	-3.81
Food type * Novel	Seeds	FALSE	TRUE	TRUE	0	two-sided	0.03	0.06	Bonferroni	-1.98

Table S3. Mixed design ANOVA for methimazole treatment and food type on food sniff rate.

Source	Sum of squares	Degrees of freedom (numerator)	Degrees of freedom (denominator)	F-values	p-values	Partial eta-squared effect sizes	Sphericity
Treatment	320.46	1	9	1.10	0.32	0.11	N/A
Food type	7807.17	1	9	39.79	0.00	0.82	TRUE
Interaction	2.92	1	9	0.01	0.91	0.00	N/A

Table S4. Mixed design ANOVA for methimazole treatment and food type on food sniff amplitude.

Source	Sum of squares	Degrees of freedom (numerator)	Degrees of freedom (denominator)	F-values	p-values	Partial eta-squared effect sizes	Sphericity
Treatment	0.02	1	9	0.07	0.79	0.01	N/A
Food type	5.12	1	9	43.12	0.00	0.83	TRUE
Interaction	0.01	1	9	0.10	0.76	0.01	N/A

Table S5. Mice used in this study.

Age (days)	Sex	Experiment	Strain
66	F	Basic paradigm	WT
88	F	Basic paradigm	WT
88	M	Basic paradigm	WT
78	M	Basic paradigm	WT
92	M	Basic paradigm	WT
93	F	Basic paradigm	WT
85	M	Basic paradigm	WT
136	M	Basic paradigm	WT
138	M	Basic paradigm	WT
107	M	Novelty, pellets → seeds	WT
107	M	Novelty, pellets → seeds	WT
107	M	Novelty, pellets → seeds	WT
81	M	Novelty, pellets → seeds	WT
81	M	Novelty, pellets → seeds	WT
81	M	Novelty, pellets → seeds	WT
70	M	Novelty, seeds → pellets	WT
70	M	Novelty, seeds → pellets	WT
70	M	Novelty, seeds → pellets	WT
91	M	Novelty, seeds → pellets	WT
91	M	Novelty, seeds → pellets	WT
91	M	Novelty, seeds → pellets	WT
76	M	Hyposmia (methimazole)	WT
77	M	Hyposmia (methimazole)	WT
100	M	Hyposmia (methimazole)	WT
100	M	Hyposmia (methimazole)	WT
100	M	Hyposmia (methimazole)	WT
100	M	Hyposmia (methimazole)	WT
124	M	Olfactory bulb stimulation (control)	WT
78	M	Olfactory bulb stimulation (control)	WT
105	M	Olfactory bulb stimulation (control)	WT
150	M	Olfactory bulb stimulation	WT
166	M	Olfactory bulb stimulation	WT
85	M	Olfactory bulb stimulation	WT
95	F	M2 silencing (control)	VGAT -
149	M	M2 silencing (control)	VGAT -
96	M	M2 silencing (control)	VGAT -
129	M	M2 silencing	VGAT +
144	F	M2 silencing	VGAT +
97	M	M2 silencing	VGAT +
111	M	M2 silencing	VGAT +
69	F	M2 silencing	VGAT +
140	F	M1 silencing	VGAT +
140	M	M1 silencing	VGAT +

125	M	M1 silencing	VGAT +
91	M	Food/fecal pellet	WT
86	M	Food/fecal pellet	WT
86	M	Food/fecal pellet	WT
96	M	Food/fecal pellet	WT
96	F	Food/fecal pellet	WT
96	M	Food/fecal pellet	WT
78	M	cross-habituation	WT
78	M	cross-habituation	WT
78	M	cross-habituation	WT
78	M	cross-habituation	WT
78	M	cross-habituation	WT
78	M	cross-habituation	WT
68	M	cross-habituation	WT
68	M	cross-habituation	WT
68	M	cross-habituation	WT
68	F	cross-habituation	WT
68	F	cross-habituation	WT
68	F	cross-habituation	WT

Supplementary Movies

Movie S1. Examples of food sniffs during oromanual food handling.
The movie shows a mouse intermittently and repeatedly bringing the food pellet under the nares in the course of handling and consuming the food. Traces show the $D_{hand\text{-}nose}$ distance, representing the kinematics of the movement, and the thermistor signal, representing the breathing signal. In the thermistor signal, inspiration and expiration appear as downward and upward trajectories, respectively. Three food sniff events are indicated by the red dots. The colored dots on the movie images are the keypoints used for markerless tracking.

Movie S2. Slowed-down examples of food sniffs.
Two 1-s segments from the food sniff events in Movie S1 are shown at slow speed, illustrating the precise coordination of the kinematics and breathing during food sniffs. For each sniff event, the time at which the $D_{hand\text{-}nose}$ distance reaches a minimum is indicated on the thermistor trace as a red dot, and the inspiratory phase of the associated breathing signal trace is colored red.

Movie S3. Contributions of head and hand movements to food sniff kinematics.
Movie segments show examples of food sniffs with relatively high, medium, and low ratios of head/hand movements.

Movie S4. Foraging and rearing.
Movie segments illustrate foraging-associated sniffing and rearing-associated sniffing behaviors.

Movie S5. Food sniffing for different food items.
Movies show food sniffing behavior for familiar pellets (left) and unfamiliar sunflower seeds (right).

Movie S6. Mouse handling a food-coated fecal pellet**.**
Movie shows a mouse handling and then rejecting a food-coated fecal pellet.

Movie S7. Food searching before and after methimazole.
Examples of food searching before and after treatment with methimazole to induce hyposmia.

Movie S8. Food sniffing after methimazole.
Examples of food sniffs after treatment with methimazole to induce hyposmia.

Movie S9. Olfactory bulb stimulation during resting or food handling.
Examples of optogenetic stimulation of the olfactory bulb during food handling and resting.

Movie S10. Suppression of food sniffing during motor cortex silencing.
Examples of optogenetic silencing of motor cortex bilaterally during food handling.
